# MRPS28 serves as a biomarker of diagnostic, prognostic, and immune modulation in pan-cancer and promotes breast cancer malignant phenotypes

**DOI:** 10.3389/fimmu.2026.1680772

**Published:** 2026-03-03

**Authors:** Xiangdong Guo, Shixing Wang, Yunlong Lu, Rensang Cai, Zhifei Luo, Jiaying Hu, Fenggui Xue, Haiyan Niu

**Affiliations:** 1 Department of Pathology, The First Affiliated Hospital, Hainan Medical University, Haikou, Hainan, China; 2 Hainan Academy of Medical Sciences, Hainan Medical University, Haikou, Hainan, China; 3 Department of Clinical Laboratory, Tianjin Medical University General Hospital Airport Hospital, Tianjin, China; 4 School of Basic Medicine and Life Sciences, Hainan Medical University, Haikou, Hainan, China; 5 Key Laboratory of Tropical Translational Medicine of Ministry of Education, School of Basic Medicine and Life Sciences, Hainan Medical University, Haikou, China

**Keywords:** MRPS28, pan-cancer, diagnostic, prognostic, breast cancer, immune infiltration

## Abstract

**Background:**

MRPS28 (Mitochondrial Ribosomal Protein S28) belongs to the MRP family and plays a critical role in mitochondrial translation and cellular energy metabolism. However, the effect of MRPS28 in pan-cancer remains unknown. This study aimed to perform a comprehensive assessment of the oncogenic potential of MRPS28 in pan-cancer using several databases, with a particular focus on breast cancer.

**Methods:**

The Cancer Genome Atlas (TCGA) and Genotype-Tissue Expression (GTEx) databases were employed to evaluate MRPS28 expression across pan-cancer. Furthermore, we analyzed the association between the expression of MRPS28 and diagnosis, prognosis, genetic alterations, genomic heterogeneity, DNA methylation, and immunity. In addition, the biological function of MRPS28 in breast cancer was investigated. The role of MRPS28 in the malignant biological behavior of breast cancer cells was studied through *in vitro* experiments, including cell proliferation, migration, and invasion.

**Results:**

Our analyses indicated that MRPS28 expression dysregulation was noted in various cancer types, and MRPS28 had remarkable diagnostic and prognostic predictive values. MRPS28 expression was substantially related to genetic alterations, genomic heterogeneity, and DNA methylation levels. In addition, MRPS28 was associated with immune infiltration and immune-related gene expression in multiple cancer types. Moreover, our experimental validation confirmed that MRPS28 knockdown considerably suppressed proliferation, migration, and invasion, as well as promoted apoptosis in the breast cancer cell lines MDA-MB-231 and MCF-7.

**Conclusion:**

Collectively, these findings imply that MRPS28 emerges as a potential diagnostic and prognostic biomarker across pan-cancer, with a potential oncogenic role in breast cancer progression. Furthermore, it may be a potential therapeutic target for various cancers.

## Introduction

Mitochondrial ribosomal proteins (MRPs) are encoded by nuclear genes and composed of a small 28S subunit and a large 39S subunit (1). MRPS28 belongs to the MRP family that differs substantially from its cytoplasmic counterparts in their composition and structure (2). Moreover, MRPS28 is located on 8q21.1-q21.2 and is a component of the small 28S subunit of the mitochondrial ribosome (mitoribosome), which is responsible for translating the 13 protein-coding genes encoded by mitochondrial DNA. These proteins are critical for the assembly and function of the oxidative phosphorylation system, ATP production, and cellular energy metabolism in eukaryotic cells (1). Proteins such as MRPS28 in mitoribosomes outnumber rRNA in proportion, thereby reflecting adaptations for specialized mitochondrial functions. MRPS28 ensures the efficient protein synthesis required for cellular energy metabolism by contributing to the mitochondrial ribosome’s stability and functionality (2). Dysregulation or mutations in MRPS28 can impair mitochondrial translation, which potentially leads to mitochondrial dysfunction and is associated with various human diseases, including cancers (1, 3, 4) and major depressive disorder (5). A previous study suggested that the dysregulation of MRPS28 expression was induced in cervical carcinoma-derived cells with radiation treatment (3). However, in bladder cancer, MRPS28 was revealed to positively regulate cell viability, migration, and invasion activity (1). Furthermore, mutations in the MRPS28 gene were involved in intrauterine growth retardation, craniofacial dysmorphism, and multisystemic involvement in patients (6). While the MRP family is well-established as key regulators of mitochondria, the specific contribution of MRPS28 to tumorigenesis is notably underexplored relative to its counterparts. This study therefore focuses on MRPS28 to elucidate its universal functions in pan-cancer using a series of tools and datasets. We investigated the relationships between MRPS28 and prognosis, diagnosis, genomic heterogeneity, DNA methylation, and immune infiltration. In addition, the gene–gene and protein–protein interaction (PPI) networks, similarly or differentially expressed MRPS28 genes, and functional enrichment analyses were performed to assess the biological function of MRPS28 in pan-cancer or breast cancer. Subsequently, we conducted various experiments to validate the biological function of MRPS28 in breast cancer cells. Based on our results, MRPS28 was revealed as a potential diagnostic and prognostic biomarker in multiple cancers and as a target for the diagnosis and treatment of breast cancer.

## Materials and methods

### Analysis of MRPS28 expression in pan-cancers

TCGA is a comprehensive cancer genomics program, while GTEx provides reference gene expression data from multiple tissues of healthy individuals. The TCGA and GTEx databases were employed to perform a systematic pan-cancer analysis of the expression of MRPS28 at the mRNA level using the SangerBox online platform (http://sangerbox.com/home.html). MRPS28 expression levels between cancer and paired adjacent normal tissues in pan-cancers from the TCGA data were analyzed using the XianTao webtool (https://www.XianTao.love/). The MRPS28 protein expression in all normal tissues and cancers was evaluated from the Human Protein Atlas database (HPA, https://www.proteinatlas.org/). Immunohistochemical images of MRPS28 in different cancers were also obtained from the HPA database. Moreover, the relationship between MRPS28 expression and the cancer types and stages of breast invasive carcinoma (BRCA) was assessed with the UALCAN database (http://ualcan.path.uab.edu/index.html). MRPS28 expression in the tumor metastasis state was explored using the TNMplot database (https://tnmplot.com/analysis/).

### Diagnostic and prognostic analyses

The SangerBox online platform was used to analyze the association between the expression of MRPS28 and the overall survival (OS), progression-free interval (PFI), disease-free interval (DFI), and disease-specific survival (DSS) of patients across several cancer types. The cutoff for significant differences in the Cox analysis was p < 0.05. The diagnostic value of MRPS28 expression was analyzed using the Sparkle online platform (https://grswsci.top), with the standard of area under the receiver operating curve (AUC) > 0.7.

### MRPS28 mutation and genomic heterogeneity analyses

Pan-cancer analyses of the frequencies of genomic mutations, amplifications, and deep deletions were performed using the cBioPortal database (https://www.cbioportal.org/). The association between the MRPS28 mRNA expression levels and copy-number variants (CNVs) was explored with the GSCALite database (https://guolab.wchscu.cn/GSCA/#/). Correlation analysis between patient survival and MRPS28 CNVs was performed in pan-cancer in the GSCALite database.

Pearson correlation between MRPS28 expression and eight tumor genomic heterogeneity indicators was analyzed using the SangerBox platform, including tumor mutation burden (TMB), mutant-allele tumor heterogeneity (MATH), microsatellite instability (MSI), neoantigen (NEO), purity, ploidy, homologous recombination deficiency (HRD), and loss of heterozygosity (LOH).

### DNA mismatch repair and stemness analyses

The data for the relationship between the MRPS28 expression and five mismatch repair (MMR) genes was obtained from the TIMER2.0 database (http://timer.cistrome.org/), and GraphPad Prism 9 software was used to analyze the data and generate the heatmap. GEPIA 2 was used to evaluate the correlation between MRPS28 expression and 29 homologous recombination repair (HRR)-related genes. Furthermore, tumor stemness scores (DNAss, RNAss, EREG.EXPss, and EREG-METHss) related to MRPS28 expression were assessed using the SangerBox online platform.

### MRPS28 DNA methylation analyses

The DNA methylation levels of MRPS28 in pan-cancer were analyzed with the UALCAN database, and the correlation between MRPS28 mRNA expression and methylation levels was analyzed using the GSCALite database. Data on the expression of five methyltransferase genes (DNMT1, DNMT3A, DNMT3B, DNMT3L, and TRDMT1) were downloaded from the TIMER2.0 database, and a heatmap was generated using GraphPad Prism 9 software. Furthermore, the association of MRPS28 methylation levels with patients’ survival probability in pan-cancer was analyzed using the MethSurv database (https://biit.cs.ut.ee/methsurv/).

### PPI network analysis and functional enrichment of MRPS28 in pan-cancers

The MRPS28-related gene–gene interaction network was created using GeneMANIA (http://genemania.org/). STRING (https://cn.string-db.org/) was used to perform the PPI network analysis of MRPS28, and the top 100 similar genes of MRPS28 were obtained from GEPIA 2. Then, the Kyoto Encyclopedia of Genes and Genomes (KEGG) database and Gene Ontology (GO) were used for functional enrichment analyses on the SangerBox platform.

### Pan-cancer analyses of MRPS28 expression in the immune microenvironment

The expression of MRPS28 was analyzed in different types of immune cells via the HPA database. Associations between MRPS28 expression and the immune subtypes and tumor-infiltrating lymphocytes across human cancers were assessed using the TISIDB database (http://cis.hku.hk/TISIDB/index.php). TIMER2.0 was employed to evaluate the correlations between MRPS28 expression and immunological characteristics, including the immune, stromal, and ESTIMATE scores. In addition, correlations between MRPS28 expression and immune-related genes in various cancers were explored using the SangerBox platform. The immune cell infiltration scores in each tumor of TCGA were evaluated based on the CIBERSORT method (7).

### LinkedOmics database analysis

The differentially expressed genes (DEG) of MRPS28 were screened from the TCGA–BRCA study in the LinkedOmics database (https://www.linkedomics.org/login.php). The Pearson correlation coefficient was presented as a volcano plot, and genes that were positively or negatively correlated with MRPS28 expression are presented in a heat map. Functional analyses were also conducted using GO terms and KEGG enrichment pathways.

### Single-cell RNA sequencing

The relationship between MRPS28 expression and 14 functional states of cancer cells was assessed using CancerSEA (http://biocc.hrbmu.edu.cn/CancerSEA/home.jsp). The distinct functional states of MRPS28 in BRCA are presented. T-SNE described the distribution of MRPS28 expression in two BRCA studies in CancerSEA. The TISCH2 (http://tisch.comp-genomics.org/) database was used to evaluate the distribution of MRPS28 expression in different immune cell types within the tumor microenvironment (TME) of BRCA according to five studies.

### Drug sensitivity analysis

The “oncoPredict” R package was used to evaluate the potential association between MRPS28 expression and drug responses (8). Samples were stratified into low and high expression groups based on the median expression of MRPS28. IC50 values of each sample, an indicator of drug sensitivity were calculated. Based on the predicted IC50 value, select drugs with high sensitivity (low IC50 value) to samples with high expression of MRPS28. Spearman correlation analysis evaluated the association between MRPS28 expression and drug sensitivity.

### Immunohistochemistry

A total of 30 pairs of BRCA paraffin tissue specimens were collected at the First Affiliated Hospital of Hainan Medical University, and the tissue arrays were made. The paraffin-embedded tissue sections (4-mm thick) were dewaxed, rehydrated, and underwent heat-induced antigen unmasking. These were then blocked with 10% goat serum and incubated with MRPS28 (1:500, A4660, ABclonal, China) primary antibodies overnight at 4°C. The sections were subsequently incubated with secondary antibody goat anti-rabbit IgG (H+L) (1:400, 31460, Thermo Fisher Scientific, USA) for 1 h at room temperature. They were then treated with diaminobenzidine, counterstained with hematoxylin, and images were captured using a Leica DM2000 light microscope (Leica, Heidelberg, Germany). All the participants from whom tissue samples were obtained provided written informed consent. The study was reviewed and approved by the Medical Ethics Committee of the First Affiliated Hospital of Hainan Medical University.

### Cell culture and transfection

The MCF-7 and MDA-MB-231 cell lines were purchased from ATCC. All cells were cultured in high-glucose Dulbecco’s Modified Eagle Medium (DMEM) containing 10% fetal bovine serum (FBS), 100 U/mL of penicillin, and 100 mg/mL of streptomycin at 37 °C under 5% CO_2_ conditions.

Cells were seeded into 12-well plates and transfected with 50 nM small interfering RNA (siRNA) targeting MRPS28 using a transfection reagent (Ribobio) when the cells reached 30%–40% confluence according to the manufacturer’s instructions. The knockdown efficiency for MRPS28 was assessed by western blot analysis at 48 h after transfection.

### Western blot analysis

Cells were lysed with RIPA lysis buffer, and the protein concentrations were determined using a BCA protein assay kit (Thermo Scientific). Thereafter, 20 µg of each protein sample was resolved by sodium dodecyl sulfate-polyacrylamide gel electrophoresis (SDS-PAGE) and transferred onto a polyvinylidene fluoride (PVDF) membrane (Millipore, Billerica, MA, USA). After incubation with 5% non-fat milk, the membranes were incubated with antibodies against MRPS28 (1:2000, A4660, ABclonal) and tubulin (1:2000, ab7291, abcam, USA) overnight at 4°C. The membranes were then incubated with horseradish peroxidase (HRP)-labeled secondary antibodies of goat anti-rabbit IgG (H+L) (1:3000, 31460, Thermo Fisher Scientific) and goat anti-mouse IgG (H+L) (catalog 31430, Thermo Fisher Scientific) for 1 h at room temperature. The blots were then visualized with an enhanced chemiluminescence (ECL) detection system.

### Transwell cell migration and invasion assays

At 48 h after transfection, the cells were harvested and resuspended in serum-free medium at a concentration of 5 × 10^5^ cells/mL. For the cell invasion assay, polyethylene terephthalate cell culture inserts with 8.0-μm pores (BD Biosciences, Franklin Lakes, NJ, USA) were placed in a 24-well culture plate. Matrigel (BD Biosciences) was added to the upper chamber, and 100 μL of the cell suspension was added to the upper chamber and incubated for 24 h at 37 °C under 5% CO_2_ conditions, and 600 μL of DMEM with 15% FBS was added to the lower chamber. After incubation, the cells on the lower surface were fixed with 4% paraformaldehyde and stained with 0.1% crystal violet. The number of cells that migrated to the lower surface was calculated. For the cell migration assay, no Matrigel was added to the upper chamber.

### Ethynyl deoxyuridine assay

Cell proliferation ability was evaluated using the Edu kit (Beyotime, C0075S, China). Briefly, cells were cultured on cover glass in 12‐well plates. At 48 h after transfection, the cells were cultured in DMEM with Edu labeling for 2 h. The cells were then fixed, permeated, and stained with the Edu antibody, and the cell nuclei were stained with labeled Hoechst 33342. Thereafter, the fluorescent signals were visualized using a fluorescence microscope (Leica DMi8, Heidelberg, Germany).

### TUNEL assay

At 48 h after transfection, the supernatant was removed, and the cells were washed with PBS. The cells were then incubated with TUNEL detection solution for 1 h, and the cell nuclei were stained with labeled Hoechst 33342. The fluorescent signals were visualized using a fluorescence microscope (Leica DMi8).

### Statistical analysis

Results are presented as the mean ± standard deviation (SD). Data were analyzed using a one-way ANOVA, and all analyses were performed using Prism software (GraphPad Software, San Diego, CA, USA). Statistical significance was set at p < 0.05.

## Results

### MRPS28 expression in pan-cancer

**Figure 1:**
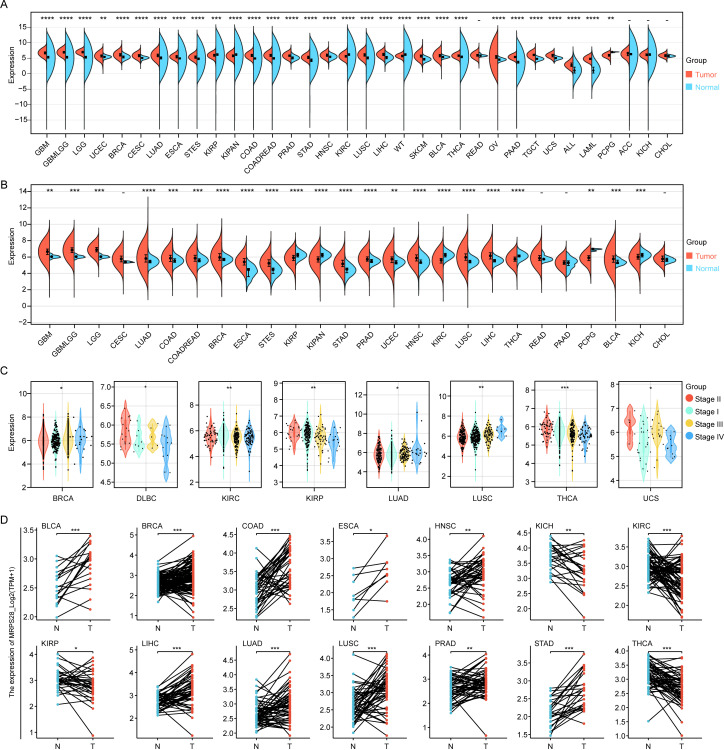
MRPS28 expression in pan-cancer. **(A)** MRPS28 expression between tumor and adjacent normal tissues in pan-cancer in the TCGA + GTEx database from SangerBox. **(B)** Pan-cancer analyses of MRPS28 expression between tumor and adjacent normal tissues in the TCGA database from SangerBox. **(C)** Expression of MRPS28 in the different tumor stages in BRCA, DLBC, KIRC, KIRP, LUAD, LUSC, THCA, and UCS. **(D)** MRPS28 expression across cancer and paired normal tissues exhibited tumor-specific expression in the TCAG database from the XianTao webtool. *p < 0.05, **p < 0.01, ***p < 0.001, and ****p < 0.0001. BRCA, breast invasive carcinoma; DLBC, diffuse large B-cell lymphoma; KIRC, kidney renal clear cell carcinoma; KIRP, kidney renal papillary cell carcinoma; LUAD, lung adenocarcinoma; LUSC, lung squamous cell carcinoma; THCA, thyroid carcinoma; UCS, uterine carcinosarcoma. 0.001.

**Figure 2:**
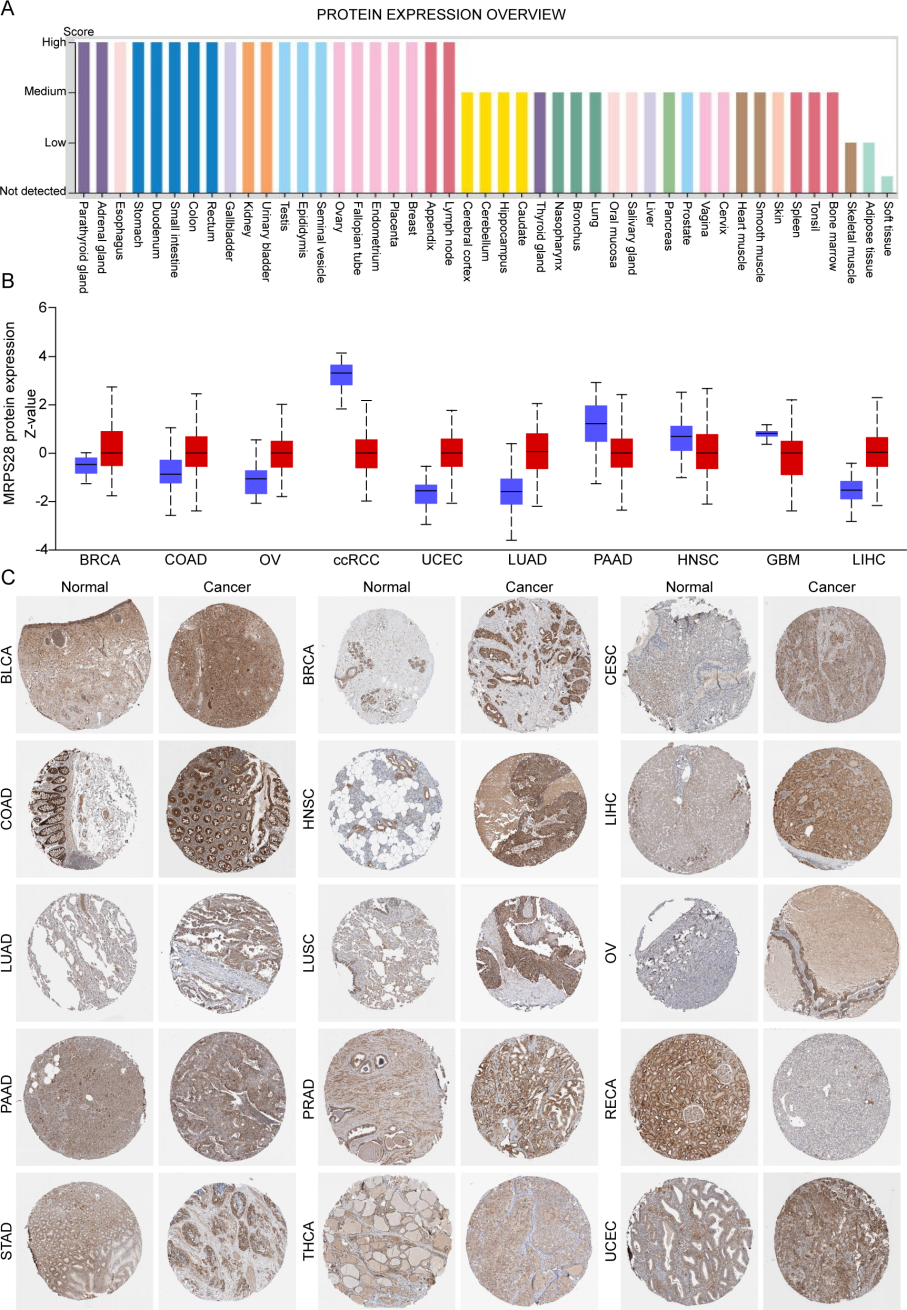
MRPS28 protein expression in different human organs and cancers. **(A)** MRPS28 expression in different human organs from the Human Protein Atlas (HPA) database. **(B)** The UALCAN database was used to analyze the protein expression of MRPS28 in normal and cancer tissues. **(C)** Immunohistochemistry images of MRPS28 expression in cancers were obtained from the HPA database.

We first evaluated the expression of MRPS28 in normal and cancer tissues in pan-cancer based on the data from TCGA and GTEx databases. We found that the expression of MRPS28 was upregulated in most cancers but was significantly decreased in kidney renal clear cell carcinoma (KIRC), kidney renal papillary cell carcinoma (KIRP), pan-kidney neoplastic disease (KIPAN), kidney chromophobe carcinoma (KICH), and Wilms tumor (**Figures 1A, B**). The correlation between MRPS28 expression and tumor stage was detected in SangerBox. We found that the MRPS28 expression was positively correlated with cancer progression in BRCA, lung adenocarcinoma (LUAD), and lung squamous cell carcinoma (LUSC), but it exhibited an opposite effect in diffuse large B-cell lymphoma (DLBC), KIRC, KIRP, thyroid carcinoma (THCA), and uterine carcinosarcoma (UCS) (**Figure 1C**). In addition, MRPS28 expression was downregulated in KICH, KIRC, and KIRP relative to that of their paired normal tissues, while it was increased in other cancers (**Figure 1D**). Next, we assessed the MRPS28 protein expression in different normal tissues (**Figure 2A**). In addition, we also analyzed the expression of MRPS28 protein in cancer. We found that the MRPS28 protein expression levels were upregulated in BRCA, colon adenocarcinoma (COAD), ovarian serous cystadenocarcinoma (OV), uterine corpus endometrial carcinoma (UCEC), LUAD, and liver hepatocellular **c**arcinoma (LIHC). However, the MRPS28 protein expression levels were downregulated in KIRC, pancreatic adenocarcinoma (PAAD), head and neck **s**quamous cell carcinoma (HNSC), and glioblastoma multiforme (GBM) (**Figure 2B**). Meanwhile, the IHC results from the HPA database indicated that there was higher MRPS28 expression in cancer tissues of bladder carcinoma (BLCA), BRCA, cervical squamous cell carcinoma and endocervical adenocarcinoma (CESC), COAD, HNSC, LIHC, LUAD, LUSC, OV, PAAD, prostate adenocarcinoma (PRAD), stomach adenocarcinoma (STAD), and UCEC, but low expression in renal cell carcinoma cancer tissues (**Figure 2C**). These findings indicate that MRPS28 expression is upregulated in most cancers but significantly downregulated in renal cancers. Its expression positively correlates with cancer progression in BRCA, LUAD, and LUSC, while showing an inverse relationship in DLBC, KIRC, and others. Protein expression analysis further supports MRPS28’s upregulation in multiple cancer tissues.

### Prognostic and diagnostic value of MRPS28 in pan-cancer

**Figure 3:**
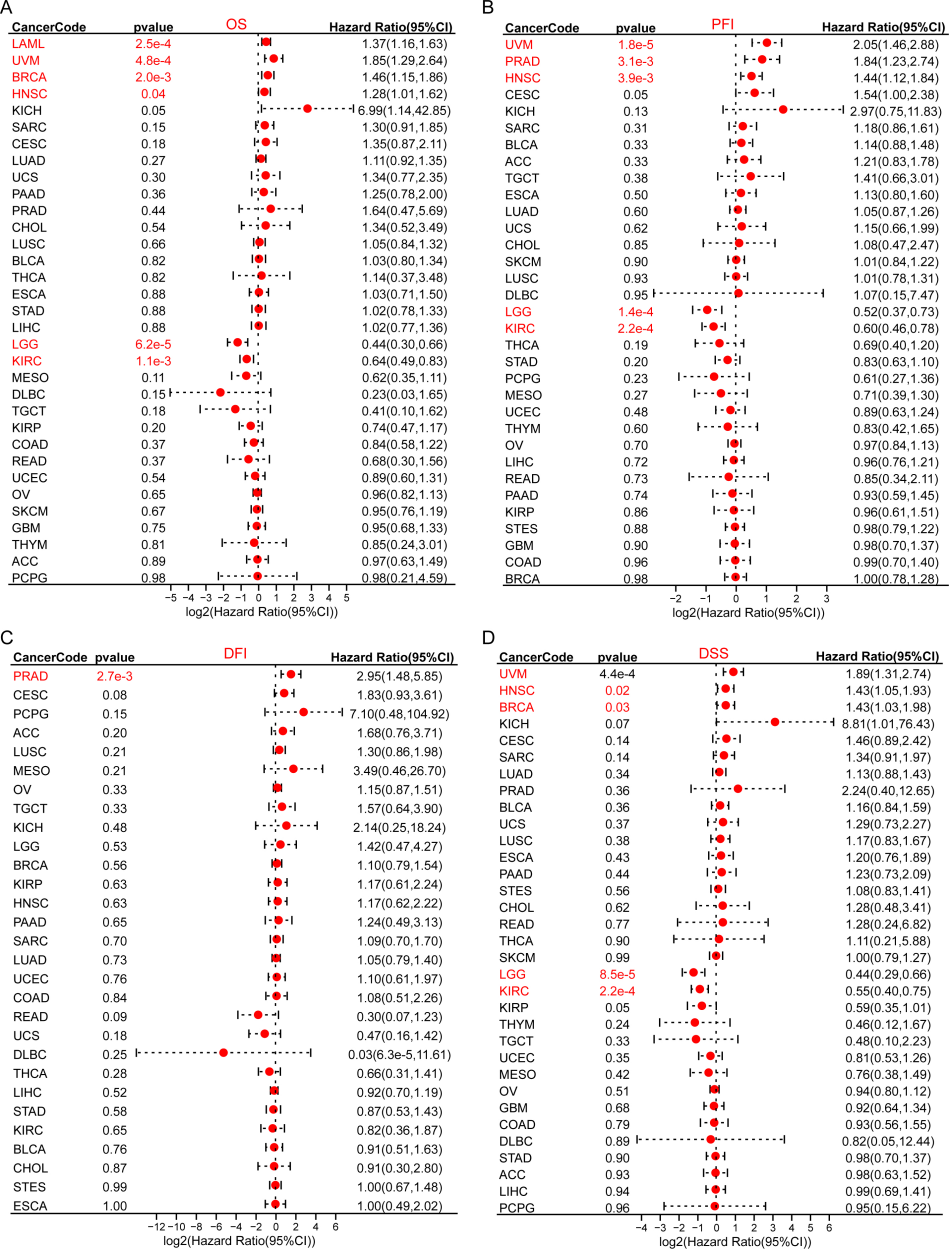
Prognostic analyses of MRPS28 in pan-cancer. Forest plots were used to analyze the relationship between MRPS28 expression and the patient’s overall survival (OS) **(A)**, progression-free interval (PFI) **(B)**, disease-free interval (DFI) **(C)**, and disease-specific survival (DSS) **(D)**.

**Figure 4:**
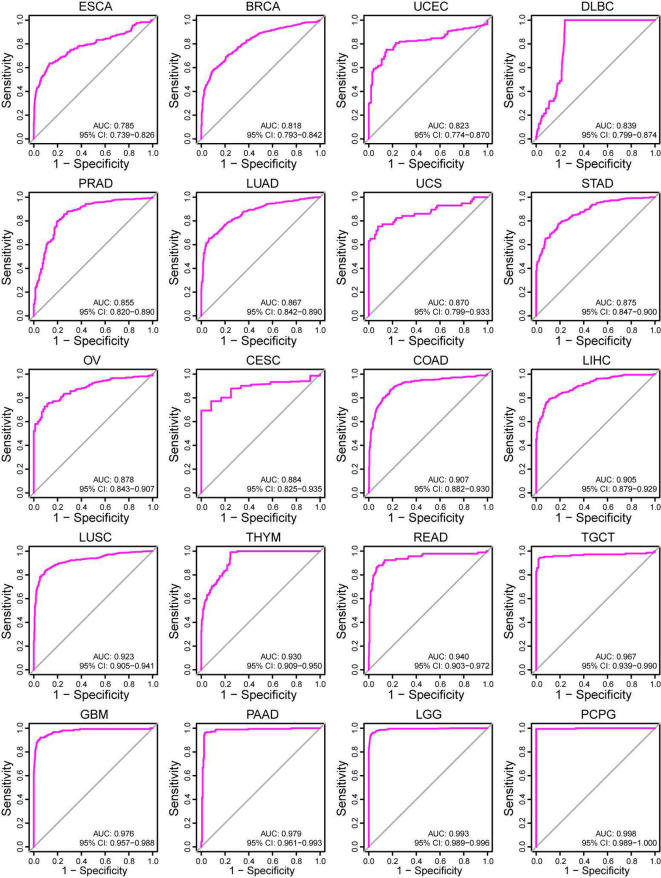
ROC curve for MRPS28 expression in pan-cancer. ROC curve of MRPS28 in pan-cancer from TCGA and GTEx databases.

The prognostic value of MRPS28 in pan-cancer was evaluated based on data from TCGA, where the survival indicators included OS, PFI, DFI, and DSS. The Cox regression analyses demonstrated that MRPS28 was significantly associated with a poorer OS in acute myeloid leukemia (LAML), uveal melanoma (UVM), BRCA, and HNSC, whereas it was associated with a better prognosis in lower-grade glioma (LGG) and KIRC (**Figure 3A**). Furthermore, MRPS28 expression was related to poor PFI in UVM, PRAD, and HNSC, while it played a protective role in LGG and KIRC (**Figure 3B**). In addition, the DFI results showed that MRPS28, as a risk factor, was associated with PRAD (**Figure 3C**). Lastly, we found that MRPS28 upregulation was correlated with a disappointing DSS in UVM, HNSC, and BRCA. Conversely, it exhibited a protective effect in LGG and KIRC (**Figure 3D**). Next, the receiver under the operator (ROC) curves based on TCGA and GTEx databases were used to assess the diagnostic value of MRPS28 in pan-cancer. MRPS28 provided a better diagnostic value in predicting the outcome when the AUC was 1. The results showed that MRPS28 had a certain accuracy (AUC > 0.7) in esophageal carcinoma (ESCA) (AUC: 0.785), BRCA (AUC: 0.818), UCEC (AUC: 0.823), DLBC (AUC: 0.839), PRAD (AUC: 0.855), LUAD (AUC: 0.867), UCS (AUC: 0.870), STAD (AUC: 0.875), OV (AUC: 0.878), CESC (AUC: 0.884), COAD (AUC: 0.907), LIHC (AUC: 0.905), LUSC (AUC: 0.923), thymoma (THYM) (AUC: 0.930), rectum adenocarcinoma (READ) (AUC: 0.940), testicular germ cell tumor (TCGT) (AUC: 0.967), GBM (AUC: 0.976), PAAD (AUC: 0.979), LGG (AUC: 0.993), and pheochromocytoma and paraganglioma (PCPG) (AUC: 0.998) (**Figure 4**). In summary, MRPS28 expression is significantly associated with prognosis and diagnostic value in various cancers, especially in BRCA, LGG, KIRC, UVM, PRAD, HNSC.

### Genetic alteration of MRPS28

**Figure 5:**
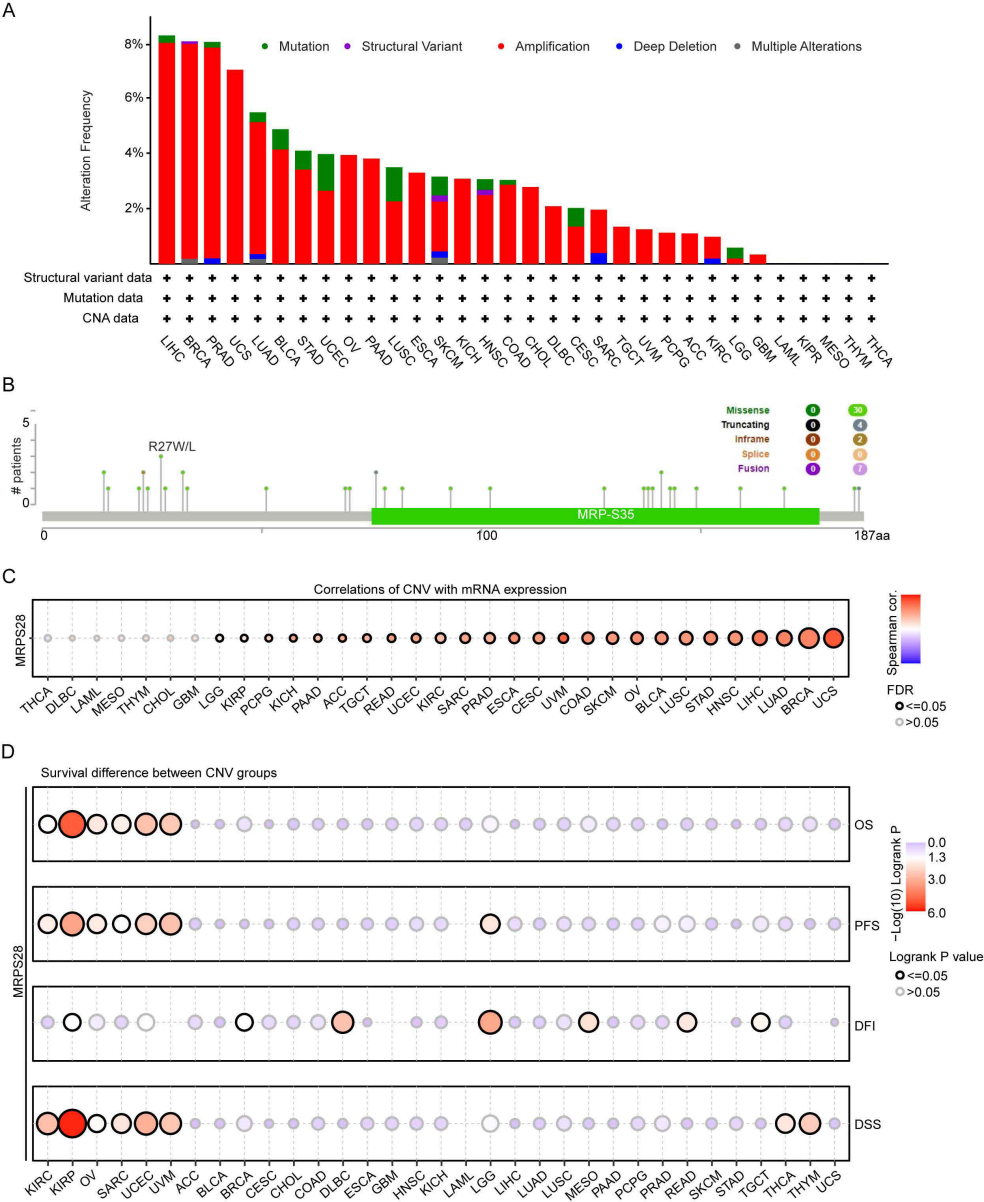
Genomic alterations and mutation prognosis of MRPS28 in pan-cancer. **(A)** The alteration frequency of MRPS28 in TCGA across human cancers using the cBioPortal database. **(B)** Lollipop diagram displaying the mutation sites of MRPS28. **(C)** Correlation between MRPS28 mRNA expression and copy number variations (CNVs) from the GSCALite database. **(D)** GSCALite database analyses indicated that the CNV of MRPS28 was related to the survival indicators overall survival (OS), progression-free interval (PFI), disease-free interval (DFI), and disease-specific survival (DSS) in pan-cancer.

Genetic alterations are often closely related to the occurrence and development of cancers (9, 10). Thus, we analyzed the MRPS28 alteration types and frequency using the cBioPortal database. The types of gene alterations included mutations, deep deletions, amplifications, structural variants, and multiple alterations. We found that amplifications accounted for the largest proportion, especially in LIHC, BRCA, PRAD, and UCS (**Figure 5A**). In addition, mutations were the second most common type of genetic alteration (**Figure 5A**). Next, the MRPS28 SNV alteration types and sites were explored using cBioPortal. We found that missense mutations of MRPS28 were the primary type of genetic alteration (**Figure 5B**). Furthermore, the R27W/L site exhibited the greatest likelihood for missense mutations in MRPS28 (**Figure 5B**). We further studied the correlation between copy number variations (CNVs) and the mRNA expression levels of MRPS28 in pan-cancer using GSCALite. The results indicated the strong correlation between CNVs and the MRPS28 mRNA expression levels among 26 cancer types (**Figure 5C**). Survival analysis revealed that CNVs of MRPS28 were related to the survival indicators of OS, PFS, DFI, and DSS in pan-cancer (**Figure 5D**), and there was a certain consistency in the prognosis among OS, PFS, and DSS (**Figure 5D**). These results suggest that genetic alterations in MRPS28 are predominantly amplifications and mutations. CNV strongly correlates with mRNA expression across cancers, and CNV status is significantly associated with patient survival.

### Genomic heterogeneity of MRPS28

**Figure 6:**
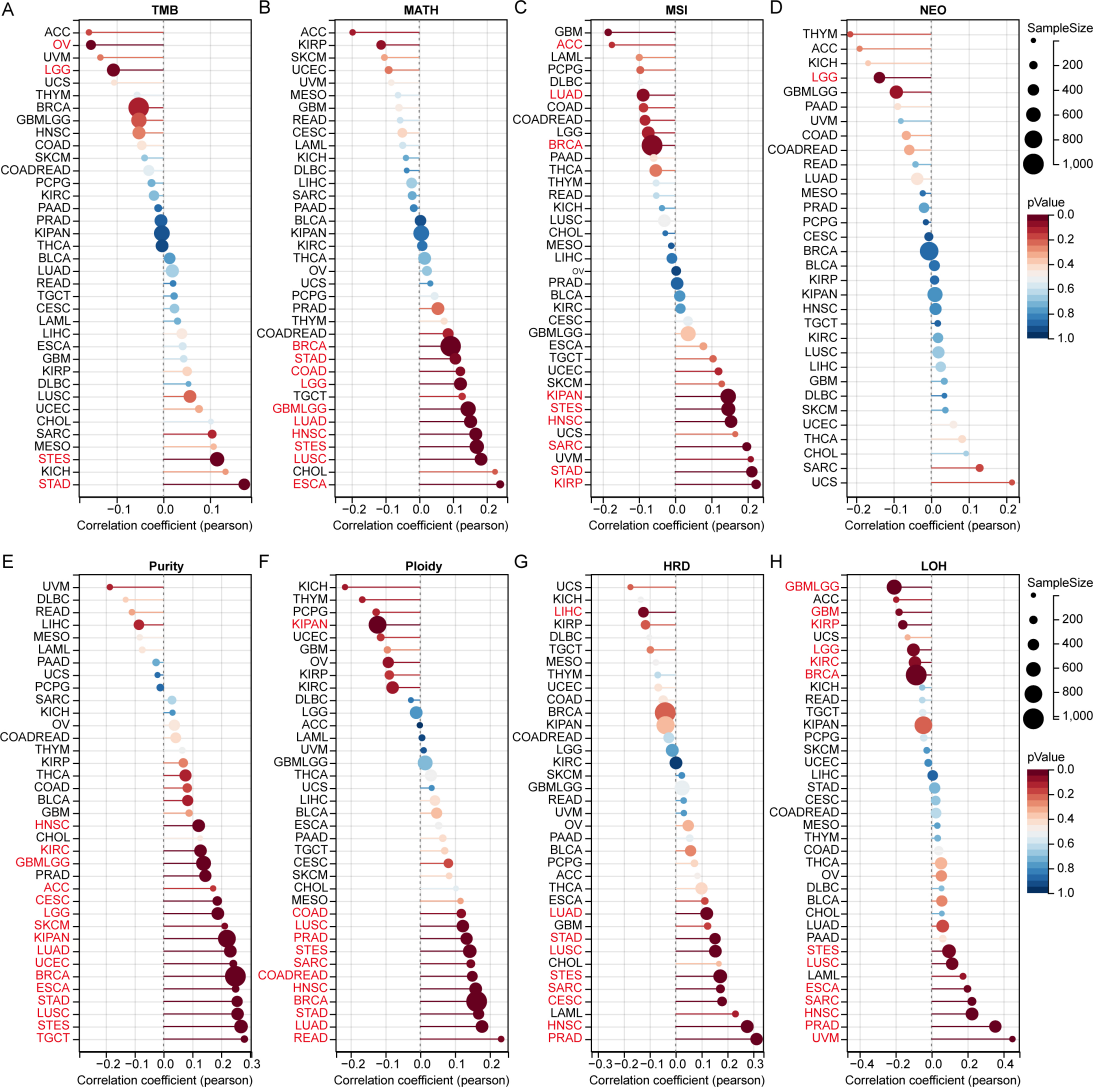
Genomic heterogeneity analyses of MRPS28 in pan-cancer. The SangerBox tool was used to assess the MRPS28 heterogeneity, including tumor mutation burden (TMB) **(A)**, mutant-allele tumor heterogeneity (MATH) **(B)**, microsatellite instability (MSI) **(C)**, neoantigen (NEO) **(D)**, purity **(E)**, ploidy **(F)**, homologous recombination deficiency (HRD) **(G),** and loss of heterozygosity (LOH) **(H)** in pan-cancer.

Tumor genomic heterogeneity was significantly correlated with prognosis, progression, and treatment failure (11, 12). Therefore, we analyzed the relationship between MRPS28 expression and genomic heterogeneity. We found the MRPS28 expression levels were positively correlated with the TMB in stomach and esophageal carcinoma (STES) and STAD, whereas they were negatively correlated in OV and LGG (**Figure 6A**). Furthermore, a positive correlation was found between the expression of MRPS28 and MATH in BRCA, STAD, COAD, LGG, glioblastoma multiforme and lower-grade glioma (GBMLGG), LUAD, HNSC, STES, LUSC, and ESCA (**Figure 6B**). The MRPS28 expression was positively correlated with MSI in KIPAN, STES, HNSC, sarcoma (SARC), STAD, and KIRP, but negatively correlated with adrenocortical carcinoma (ACC), LUAD, and BRCA (**Figure 6C**). There was a negative relationship between MRPS28 expression and NEO in LGG (**Figure 6D**). Furthermore, MRPS28 expression was positively correlated with purity in HNSC, KIRC, GBMLGG, ACC, CESC, LGG, skin cutaneous melanoma (SKCM), KIPAN, LUAD, UCEC, BRCA, ESCA, STAD, LUSC, STES, and TCGT (**Figure 6E**). A positive relationship was detected between MRPS28 expression and ploidy in COAD, LUSC, PRAD, STES, SARC, colon adenocarcinoma and rectum adenocarcinoma (COADREAD), HNSC, BRCA, STAD, LUAD, and READ, and a negative correlation in KIPAN (**Figure 6F**). In addition, a positive correlation was identified between MRPS28 and HRD in LUAD, STAD, LUSC, STES, SARC, CESC, HNSC, and PRAD, whereas a negative relatio nship was discovered in LIHC (**Figure 6G**). Finally, a positive relationship was found between MRPS28 and LOH in STES, LUSC, ESCA, SARC, HNSC, PRAD, and UVM, but negatively correlated with GBMLGG, GBM, KIRP, LGG, KIRC, and BRCA (**Figure 6H**). Thus, MRPS28 expression correlates with various tumor genomic heterogeneity indicators, suggesting a potential role in regulating genomic stability and heterogeneity.

### MRPS28 is related to DNA repair and tumor stemness

**Figure 7:**
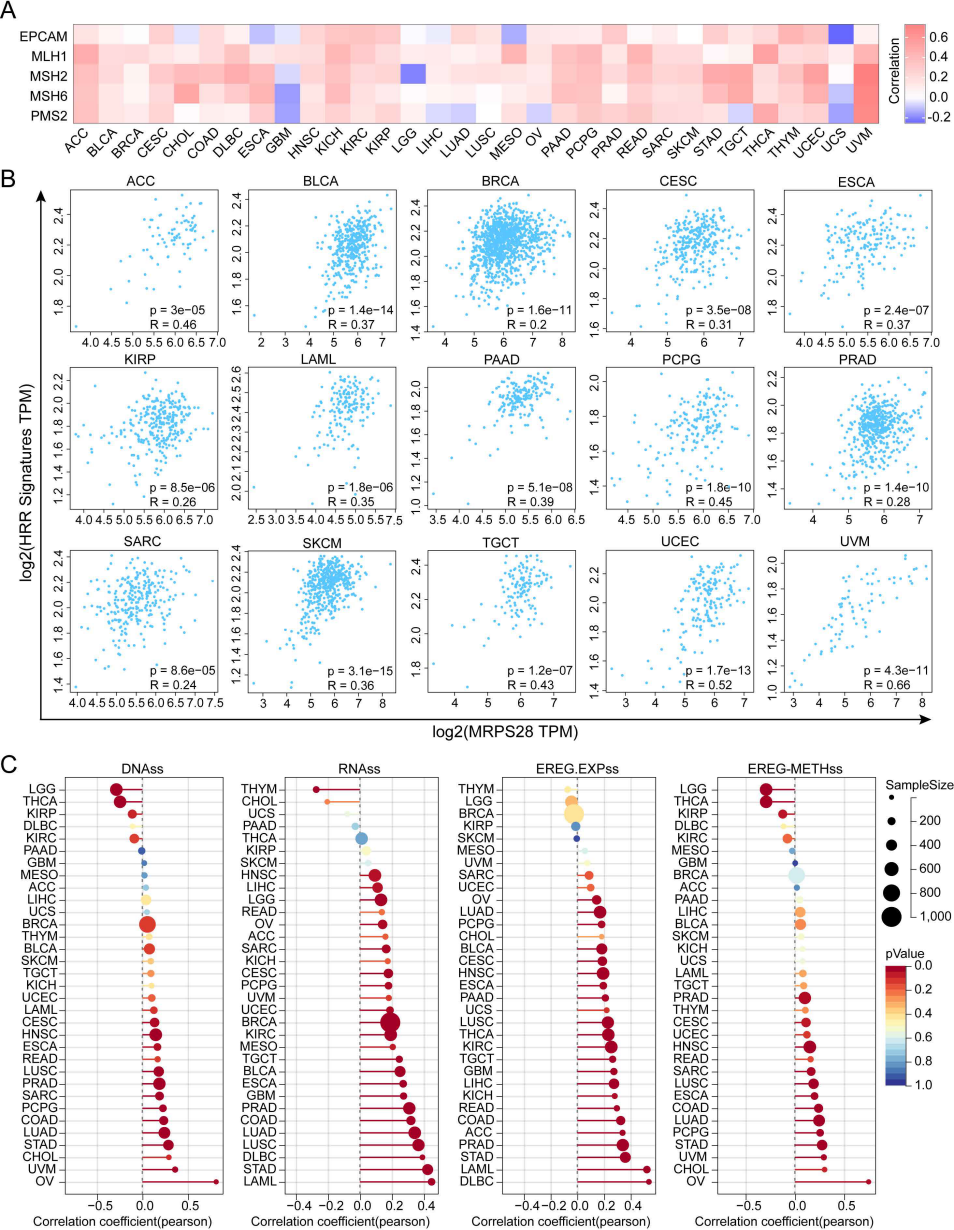
Correlation between MRPS28 expression and DNA repair and stemness. **(A)** The TIMER2.0 database was used to explore the correlation between MRPS28 and five mismatch repair (MMR) genes (EPCAM, MLH1, MSH2, MSH6, and PMS2) in pan-cancer, and a heatmap is presented. **(B)** Correlation scatter plots between MRPS28 and 29 homologous recombination repair (HRR) gene signatures in the GEPIA 2 database. **(C)** MRPS28 was related to tumor stemness via SangerBox analysis.

DNA damage responses are complex processes that are vital for maintaining the cellular genome’s stability and integrity by screening and removing abnormal sequences or structures within chromosomes (13), including the MMR and HRR pathways (14). The HRR and MMR pathways can help tumor cells evade certain treatment methods, which can enable these tumor cells to acquire stemness (15). We first evaluated the association between the MRPS28 and MMR genes. The results indicated that there was a significant and positive correlation between MRPS28 expression and MMR genes in more than 22 tumors. Importantly, apart from GBM and UCS, most MMR genes were positively correlated with the expression of MRPS28 (**Figure 7A**). Furthermore, MRPS28 expression was positively correlated with HRR signatures in ACC, BLCA, BRCA, CESC, ESCA, KIRP, LAML, PAAD, PCPG, PRAD, SARC, SKCM, TGCT, UCEC, and UVM (**Figure 7B**).

Cancer stem cells (CSCs) are capable of self-renewing and can promote tumor progression by inducing growth, metastasis, and drug resistance (16). Four stemness indexes, including DNAss, RNAss, EREG.EXPss, and EREG-METHss, were selected based on mRNA expression and DNA methylation. The association between MRPS28 and the cancer stemness indexes in pan-cancer was then evaluated. The results demonstrated that MRPS28 was positively correlated with cancer stemness indexes in most cancer types (**Figure 7C**). In summary, MRPS28 expression positively correlates with DNA damage repair genes and cancer stemness indices in most cancers, indicating its possible involvement in promoting tumor progression through DNA repair and stemness regulation.

### DNA methylation of MRPS28 in pan-cancer

**Figure 8:**
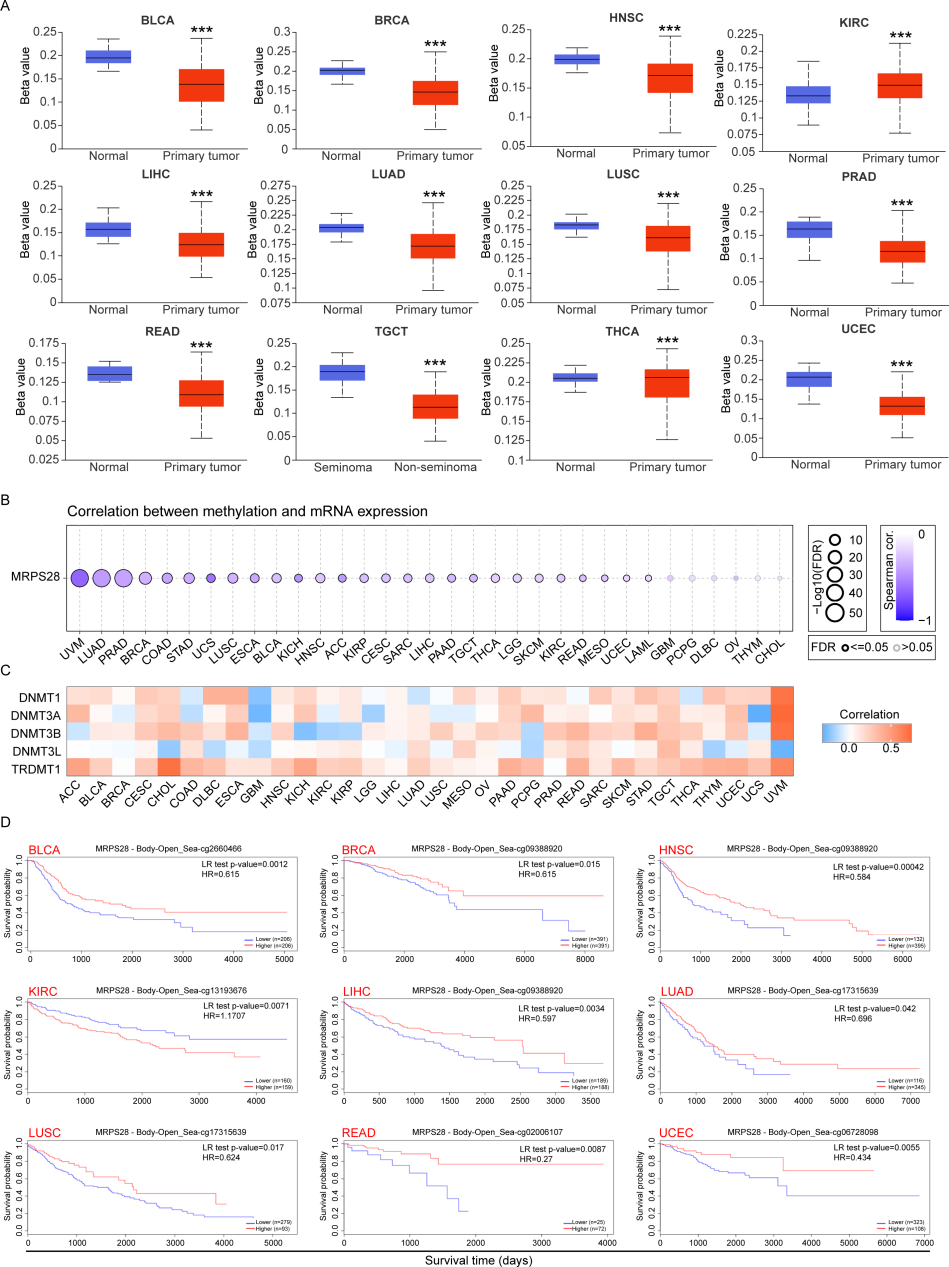
DNA methylation analyses of MRPS28 in pan-cancer. **(A)** The DNA methylation levels of MRPS28 were evaluated using the UALCAN database. **(B)** Correlations between MRPS28 mRNA expression and its DNA methylation levels were assessed in GSCALite. **(C)** The relationship between MRPS28 expression and DNA methyltransferase, including DNMT1, DNMT3A, DNMT3B, DNMT3L, and TRDMT1, across cancer types is shown. **(D)** DNA methylation levels of MRPS28 were related to the prognosis of patients with cancer. ***p < 0.001.

DNA methylation also contributes to the development of cancers by regulating gene expression (17, 18). The levels of MRPS28 methylation were analyzed using the UALCAN database (**Figure 8A**). We found that the MRPS28 methylation levels were significantly decreased in BLCA, BRCA, HNSC, LIHC, LUAD, LUSC, PRAD, READ, TGCT, and UCEC, whereas the higher levels of MRPS28 methylation were detected in KIRC and THCA (**Figure 8A**). Methylation levels usually affect the mRNA expression of genes. Thus, we further explored the association between MRPS28 methylation and its mRNA expression using the GSCALite database. The results indicated that the MRPS28 methylation levels were negatively related to the MRPS28 mRNA levels in pan-cancer (**Figure 8B**). DNA methylation is catalyzed by DNA methyltransferases (DNMTs), which control the growth, cell cycle, differentiation, migration, invasion, and death characteristics of cancer cells. We found that the expression of MRPS28 was significantly positively correlated with DNMTs levels in BLCA, CESC, HNSC, OV, PAAD, PRAD, SARC, SKCM, STAD, and TGCT (**Figure 8C**). Based on these findings, we assessed the correlation between MRPS28 methylation and patients’ prognosis. The results demonstrated that patients exhibited a better prognosis with higher levels of MRPS28 methylation in BLCA, BRCA, HNSC, LIHC, LUAD, LUAC, READ, and UCEC. However, their prognosis was poor in KIRC with lower levels of MRPS28 methylation (**Figure 8D**). These results suggest that MRPS28 methylation related epigenetic regulation is also involved in the progression of cancers.

### Enrichment analysis of MRPS28-related genes

**Figure 9:**
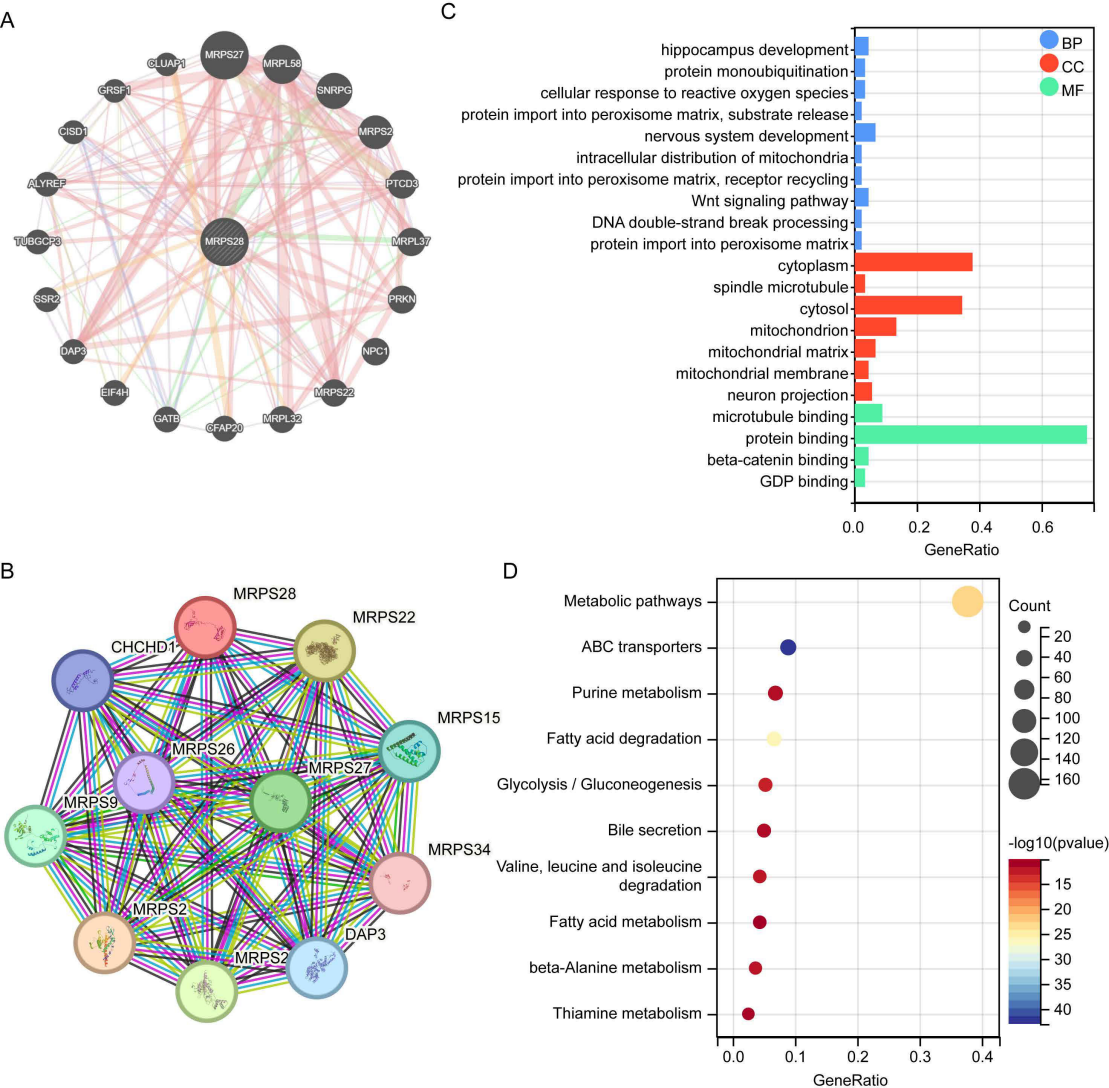
Analyses of gene–gene and protein–protein network, differentially expressed genes (DEGs), Gene Ontology (GO), and Kyoto Encyclopedia of Genes and Genomes (KEGG) enrichment. **(A)** The interaction networks of the MRPS28-related gene–gene interactions via GeneMANIA. **(B)** The protein–protein interaction (PPI) network of MRPS28 and its interacting proteins was obtained from the STRING database. **(C)** GO analysis of the top 100 similar genes of MRPS28. **(D)** KEGG analysis of the top 100 similar genes of MRPS28.

The interaction networks of the MRPS28-related gene–gene were created via GeneMANIA and STRING (**Figures 9A, B**). To explore the biological functions of MRPS28, we first screened the similar genes of MRPS28 from the GEPIA 2 database and then performed KEGG and GO enrichment analyses to explore the functions of the identified similar genes. The GO analysis results indicated that similar genes of MRPS28 were primarily related to protein and microtubule binding (**Figure 9C**). The KEGG pathway enrichment analysis indicated that similar genes of MRPS28 were primarily involved in metabolic pathways, ABC transporters, purine metabolism, and fatty acid degradation (**Figure 9D**). The rare biological functions of MRPS28 may be related to the insufficient study of MRPS28-related genes. At least partially explain that MRPS28-associated genes are primarily involved in protein binding, microtubule binding, purine metabolism, and fatty acid degradation, highlighting its potential role in cellular metabolism.

### Correlation between MRPS28 expression and immune characteristics

**Figure 10:**
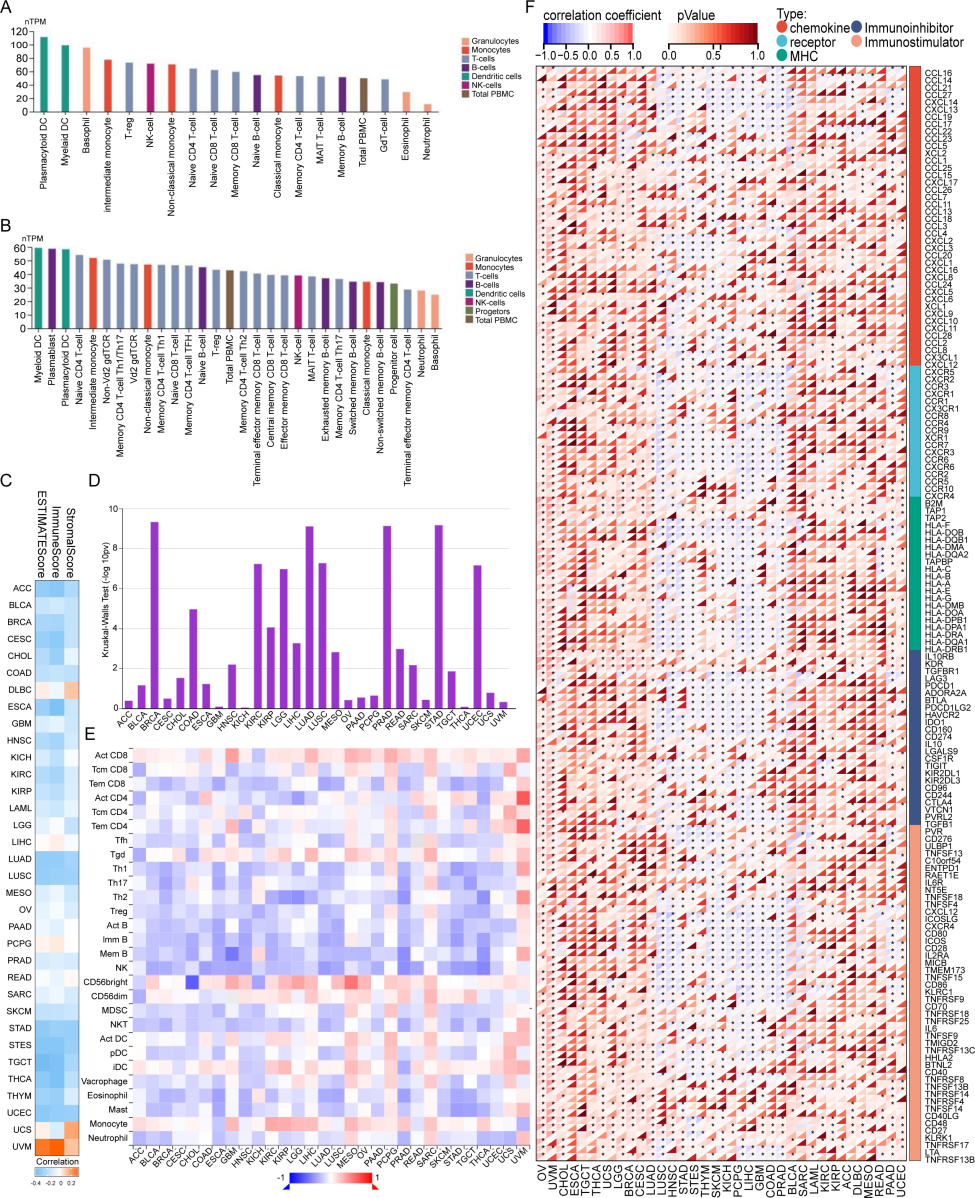
Analysis of the relationship between MRPS28 and immune characteristics. The expression of MRPS28 in different types of immune cells from the Human Protein Atlas (HPA) **(A)** and Monaco datasets **(B)**. **(C)** Correlations between the MRPS28 expression levels and stromal, immune, and ESTIMATE scores are presented. **(D)** Correlations between MRPS28 and immune subtypes from the TSIDB database. **(E)** MRPS28 expression levels were related to the infiltration of immune cells from the TSIDB database. **(F)** Correlation analyses between MRPS28 and chemokines, immune receptors, major histocompatibility complexes (MHCs), immunoinhibitors, and immunostimulators.

The immune TME plays an important role in cancer progression, metastasis, and treatment (19, 20). Elucidating the multifaceted roles and dynamic regulatory mechanisms of target genes or other biological events on the tumor immune microenvironment can help develop novel immunotherapy targets and potentially optimize existing treatment strategies (21–23). To explore the role of MRPS28 in the immune TME across various cancer types, we assessed the correlation between MRPS28 expression and immune cell infiltration, immune scores, and the expression of immune-related genes in cancers. We found that MRPS28 was widely expressed in various immune cell types via the HPA and Monaco datasets (**Figures 10A, B**). Data from TIMER2.0 revealed that MRPS28 expression was negatively correlated with stromal, immune, and ESTIMATE scores in most cancers (**Figure 10C**). The TISIDB database was used to analyze the relationship between MRPS28 expression and the immune subtypes in pan-cancer. The results indicated that MRPS28 expression was significantly associated with specific immune subtypes (**Figure 10D**). We further identified that there was a greater negative correlation between MRPS28 expression and the infiltration of immune cells, including effector memory CD8, Th1, Th2, Th17, Treg, activated B cells, memory B cells, immature B cells, and natural killer (NK) T cells in most cancers (**Figure 10E**). The relationship between the MRPS28 expression levels and chemokines, immune receptors, major histocompatibility complexes (MHCs), immunoinhibitors, and immunostimulators was detected from the SangerBox database. We found that the expression of MRPS28 was positively associated with these immune indicators in OV and UVM, whereas a negative relationship was detected in LUSC, HNSC, STAD, STES, THYM, SKCM, KICH, PCPG, LIHC, GBM, COAD, and PRAD (**Figure 10F**). Last, we evaluated the relationship between MRPS28 expression and infiltration scores of 22 immune cells in 33 cancer types by CIBERSORT (**Supplementary Figure 1**). Notably, MRPS28 expression was highly positively correlated with M2 macrophage and activated mast cell, but negatively correlated with Treg, gamma delta T cell, CD8^+^T cell, and cell memory B in most of the 33 types of cancer, especially in BRCA, KIRC, COAD, HNSC, THCA, STAD. These results suggested that MRPS28 is expressed in various immune cells and correlates negatively with immune and stromal scores in the tumor microenvironment. Its expression is also linked to immune cell infiltration and immune-related gene expression, suggesting a role in tumor immune regulation.

### Expression and function of MRPS28-related genes in BRCA

**Figure 11:**
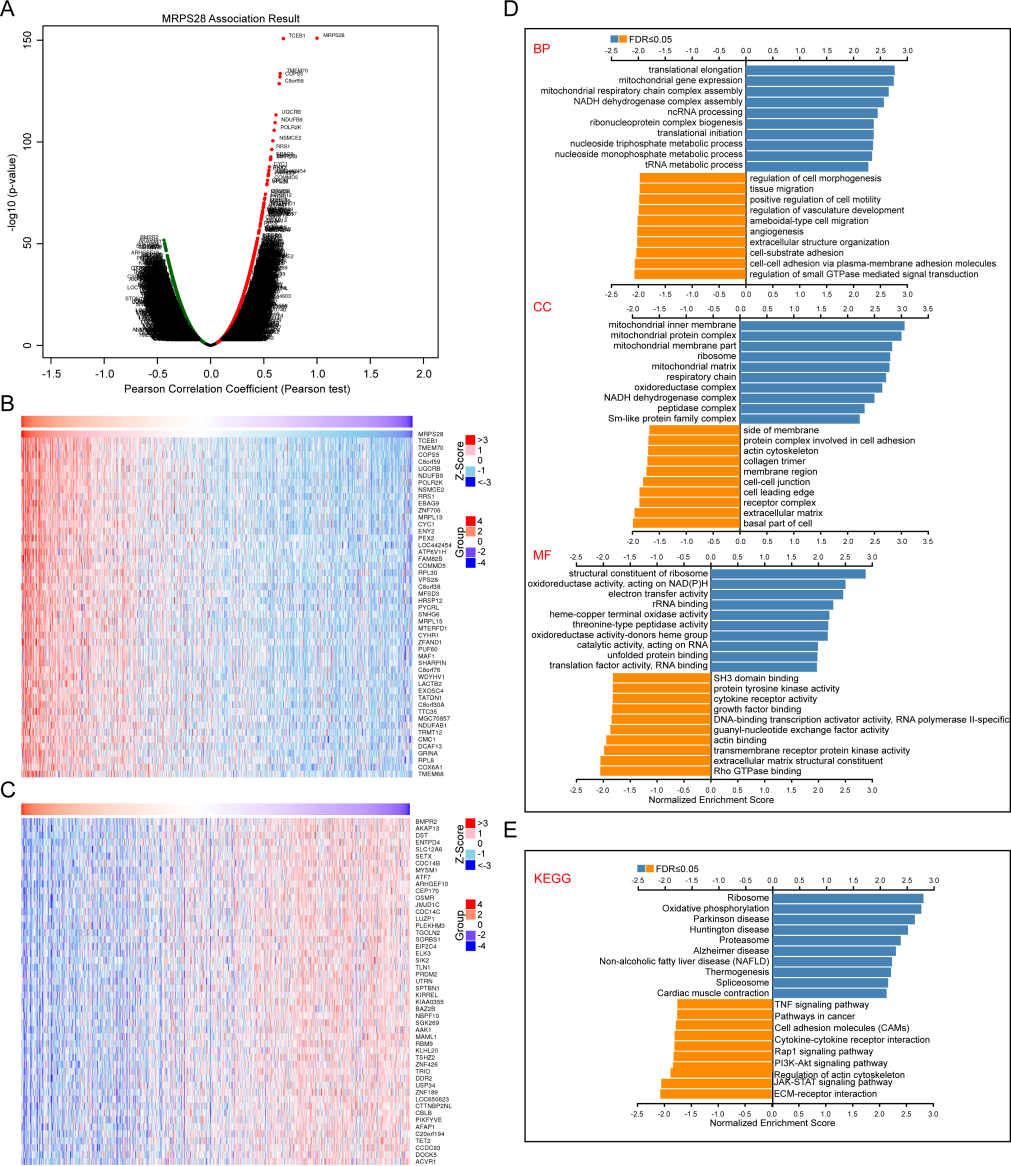
Coexpression of MRPS28 genes and their functional analyses in breast invasive carcinoma (BRCA) using the LinkedOmics database. **(A)** Volcano plots showing the MRPS28 genes that were significantly related to BRCA, as distinguished by the Pearson test; left: negatively related with MRPS28, right: positively related with MRPS28. The top 50 genes that were positively **(B)** and negatively **(C)** related to MRPS28 in BRCA. Gene Ontology (GO) annotation **(D)** and Kyoto Encyclopedia of Genes and Genomes (KEGG) pathway **(E)** analyses of the MRPS28-related genes in BRCA.

We further evaluated the potential biological function of MRPS28 in BRCA using the LinkedOmics database. The volcano map shows genes that are positively (right, red dots) or negatively (left, green dots) correlated with MRPS28 expression (**Figure 11A**). The heat maps include the top 50 genes that are positively or negatively associated with MRPS28 expression (**Figures 11B, C**). In addition, we analyzed the biological function of MRPS28 in BRCA. GO analysis revealed that the MRPS28-related genes are mainly enriched in the structural constituent of ribosomes, oxidoreductase activity, acting on NAD(P)H, electron transfer activity, rRNA binding, heme-copper terminal oxidase activity, threonine-type peptidase activity, oxidoreductase activity-donors heme group, catalytic activity, acting on RNA, unfolded protein binding, translation factor activity, and RNA binding (**Figure 11D**). In addition, KEGG pathway analysis indicated enrichment in the ribosome, oxidative phosphorylation, Parkinson’s disease, Huntington’s disease, proteasome, Alzheimer’s disease, non-alcoholic fatty liver disease, thermogenesis, spliceosome, and cardiac muscle contraction pathways (**Figure 11E**). In a word, MRPS28 expression is associated with genes enriched in ribosome structure, oxidative phosphorylation, and RNA binding in breast cancer,.

### Correlation between MRPS28 expression and the functional states and immunity in breast cancer

**Figure 12:**
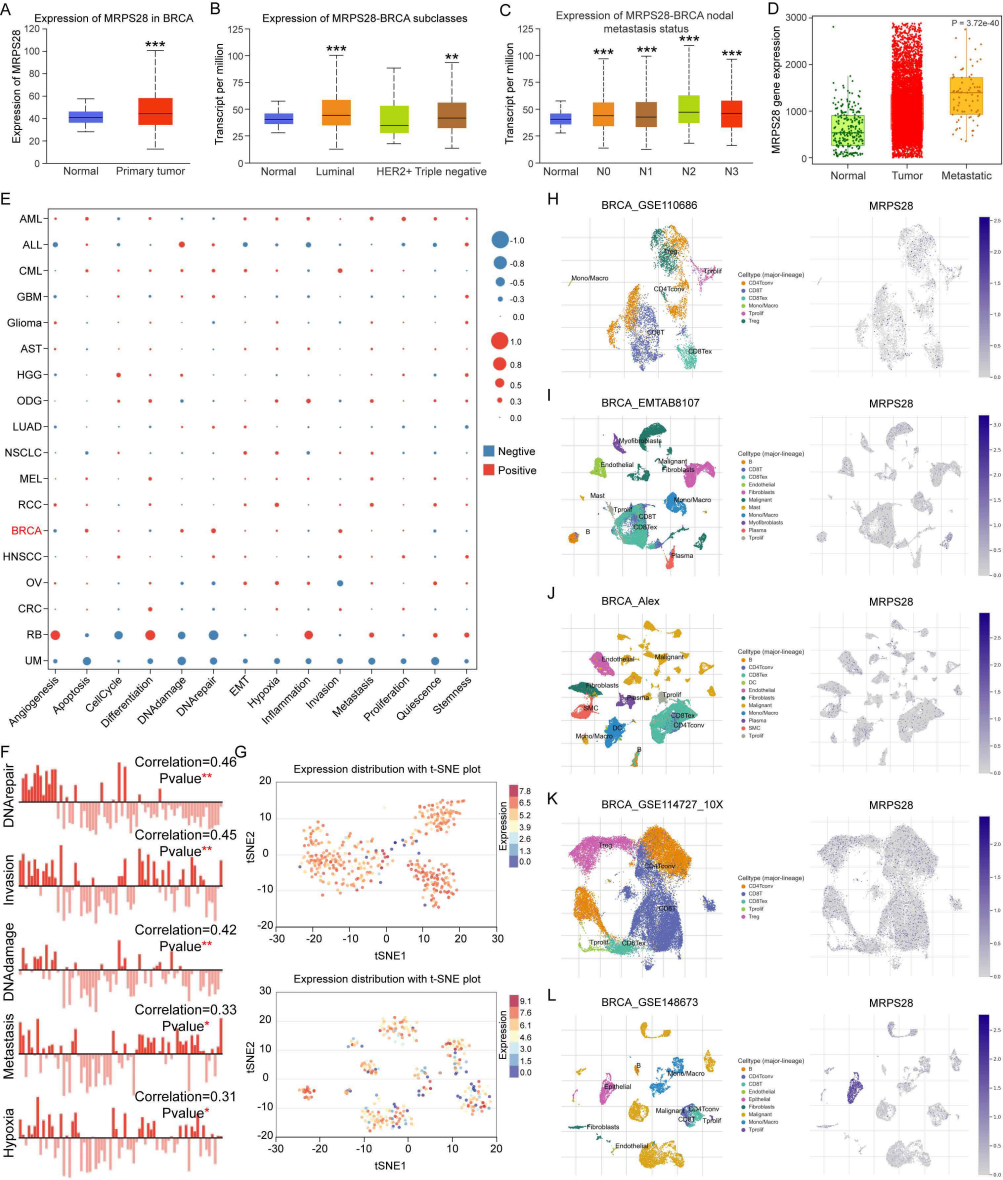
Correlation between MRPS28 expression and the functional states and immunity in breast invasive carcinoma (BRCA). **(A)** MRPS28 expression in normal and cancer tissues of patients with BRCA from the UALCAN database. **(B)** MRPS28 expression in normal and various types of BRCA tissues. **(C)** Based on cancer stage in patients with BRCA, the expression of MRPS28 was assessed using the UALCAN database. **(D)** MRPS28 expression in normal tissue and non-metastatic and metastatic tissue from patients with cancer. **(E)** Correlation between MRPS28 and 14 functional states in pan-cancer. **(F)** MRPS28 expression was related to DNA repair, invasion, DNA damage, metastasis, and hypoxia in BRCA. **(G)** MRPS28 expression distribution is shown via t-SNE in BRCA. **(H–L)** Single cell sequencing results revealed the MRPS28 expression distribution in different immune cells of the tumor microenvironment (TME) in BRCA. **p < 0.01 and ***p < 0.001.

Data from TCGA in UALCAN further indicated that MRPS28 expression was increased in BRCA (**Figure 12A**). The MRPS28 expression levels were upregulated in luminal and triple-negative BRCA subtypes but not in HER2+ breast cancer (**Figure 12B**), but not in HER2+ breast cancer (**Figure 12B**). Furthermore, the high expression of MRPS28 was related to the lymph node metastasis (**Figure 12C**). Metastasis is the main cause of death in patients with BRCA. Therefore, we detected the expression of MRPS28 in patients with non-metastatic or metastatic BRCA. The results demonstrated that the expression of MRPS28 was higher in patients with metastatic BRCA than in patients with non-metastatic BRCA (**Figure 12D**). The effects of MRPS28 expression on the functional states of breast cancer cells were then assessed using CancerSEA. We found that MRPS28 expression was significantly related to the different functional states, especially in acute lymphoblastic leukemia (ALL), retinoblastoma (RB), and uveal melanoma (UM) (**Figure 12E**). In BRCA, we primarily focused on the positive relationship between MRPS28 expression and DNA repair, invasion, DNA damage, metastasis, and hypoxia (**Figure 12F**). Furthermore, the single-cell RNA sequencing results indicated that MRPS28 was widely distributed in BRCA cells (**Figure 12G**). According to the TISCH2 database, the single-cell RNA sequencing data demonstrated that MRPS28 expression was also distributed in many types of immune cells in the TME of BRCA (**Figures 12H–L**). Interestingly, compared to other types of cancer, CIBERSORT scores indicated that the expression of MRPS28 is significantly correlated with the infiltration of multiple immune cells in BRCA (**Supplementary Figure 1**). Importantly, we found that the expression of MRPS28 was positively correlated with the infiltration of M2 macrophage, resting NK cells, and activated mast cells, but negatively correlated with CD8^+^T cell, gamma delta T cell, follicular helper T cell, resting memory CD4^+^T cell, activated NK cell, resting myeloid dendritic cell, M1 macrophage, and naive B cells in BRCA. These results suggest that MRPS28 is highly expressed in breast cancer, particularly in luminal and triple-negative subtypes, and correlates with lymph node metastasis and distant metastasis. Increased MRPS28 expression may facilitate the formation of an immunosuppressive tumor microenvironment and lead to immune escape of tumor cells in BRCA.

### MRPS28 knockdown impeded the malignant biological behavior of BRCA cells

**Figure 13:**
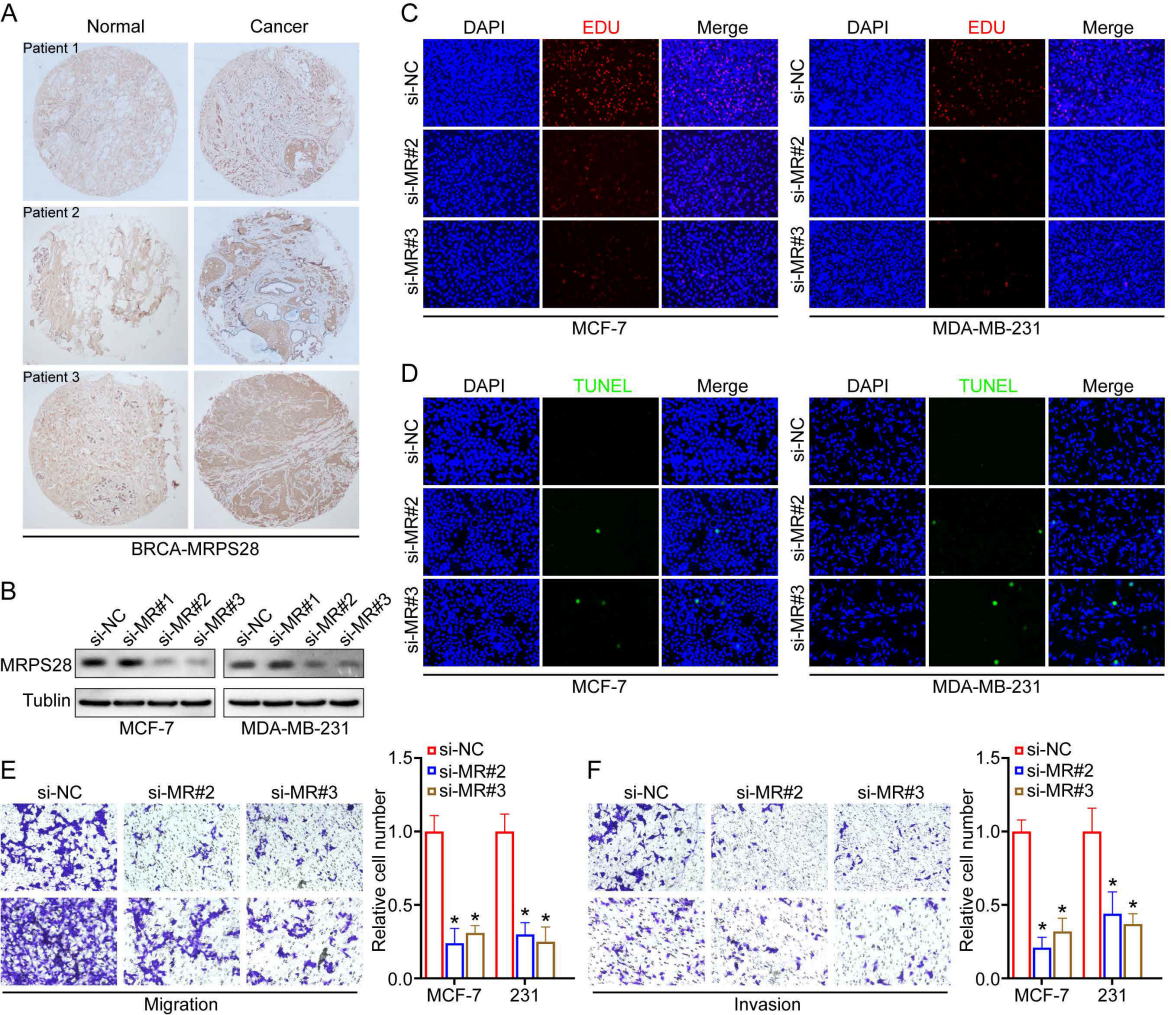
MRPS28 knockdown inhibited the malignant biological behavior of breast invasive carcinoma (BRCA) cells. **(A)** Representative immunohistochemistry images of MRPS28 in the adjacent normal tissues and cancer tissues of BRCA. **(B)** Western blot analysis was used to detect the knockdown efficiency of MRPS28 in MCF-7 and MDA-MB-231 cells. **(C)** Cell proliferation ability was assessed using ethynyl deoxyuridine (Edu) assays. **(D)** TUNEL assays were used to detect cell apoptosis. The migration **(E)** and invasion **(F)** abilities of MCF-7 and MDA-MB-231 cells with MRPS28 knockdown were evaluated with transwell assays. *p < 0.05.

Based on the above results, we hypothesize that MRPS28 might regulate the fate of BRCA cells. We collected 30 pairs of clinical specimens from patients with BRCA and used IHC to detect the MRPS28 protein expression. The IHC results indicated that MRPS28 was highly expressed in cancer tissues compared with that of their paired normal tissue counterparts (**Figure 13A**). Then, we knocked down MRPS28 with siRNA in breast cancer cell lines (MCF-7 and MDA-MB-231) to further investigate its effect on the malignant biological behavior of cancer cells. Si-MRPS28#2 and si-MRPS28#3 were selected for the subsequent assays because of their greater knockdown efficiency (**Figure 13B**). We found that MRPS28 knockdown inhibited the proliferative ability of MCF-7 and MDA-MB-231 using EDU assays (**Figure 13C**). Furthermore, TUNEL assays indicated that MRPS28 knockdown promoted cancer cell apoptosis (**Figure 13D**). In addition, MRPS28 knockdown significantly reduced the cancer cells’ migration and invasion abilities (**Figures 13E, F**). Overall, these cell experiments further support the carcinogenic effect of MRPS28 in breast cancer.

### Drug sensitivity analysis

Screening for potential drugs that target MRPS28 is crucial for developing new therapies. Drug sensitivity analysis, performed using the “oncoPredict” R package, revealed a significant positive correlation between MRPS28 expression and sensitivity to some drugs in BRCA. According to the ranking of correlation coefficient values from high to low, we present the following 9 drugs, SB505124, AZD6482, Tozasertib, ZM447439, Staurosporine, AZD8055, Dasatinib, GSK269962A, and Foretinib (**Supplementary Figure 2**).

## Discussion

Our study systematically elucidated the key role of MRPS28 in pan-cancer and breast cancer. Here, we found that MRPS28’s upregulation in most cancers was significantly associated with poor prognosis and demonstrates excellent diagnostic value. The expression of MRPS28 may be co-regulated by gene amplification and promoter hypomethylation, and closely linked to tumor genomic heterogeneity, DNA damage repair capacity, and cancer stemness. Further research revealed that MRPS28 is broadly involved in regulating the tumor immune microenvironment, with its expression correlated to immune cell infiltration, immune checkpoint molecule and other immune-related genes expression. Finally, functional validation focused on breast cancer confirms that MRPS28 regulated the proliferation, migration, invasion, and apoptosis of breast cancer cell. These findings clarified that MRPS28 promotes tumor progression through multi-dimensional mechanisms, highlighting its potential as a cross-cancer or breast cancer prognostic biomarker and therapeutic target.

Previous studies have shown that MRPS28, as a pro-cancer factor, is closely related to the progression of several cancers. For example, the splice variant kDec03 expression of the MRPS28 gene was altered during the process of radiation treatment for the cervical carcinoma-derived cell line SiHa, which may be related to the radiation sensitivity of SiHa cells (3). MRPS28 accelerated the progression of bladder cancer in response to centromere protein U (1). In addition, benzyl isothiocyanate treatment decreased cell viability and induced cell apoptosis in GBM 8401 cells by altering the expression of a series of key genes, including MRPS28 (4). These results suggest that MRPS28 may have potential oncogenic effects.

In this study, first, we investigated MRPS28 expression at the transcriptional and translational levels in various databases. We found that MRPS28 expression was upregulated in many cancers compared with their adjacent normal tissues, while its expression was notably reduced in specific cancers such as KIRC, KIRP, and other renal-related tumors. These findings suggest that MRPS28 may play distinct roles across different cancers, thus potentially reflecting tumor-specific regulatory mechanisms. In BRCA, the expression of MRPS28 increases at both mRNA and protein levels, and is positively correlated with the patient’s clinical condition. Furthermore, to confirm whether MRPS28 could be employed as a prognostic biomarker, we analyzed the prognostic and diagnostic value of MRPS28. Notably, our results revealed that the OS (eight cancers), PFI (five cancers), DFI (one cancer), and DSS (five cancers) were associated with MRPS28 expression. However, MRPS28 played a dual role in the prognosis of patients across a few cancer types. These findings emphasize the complex and context-dependent role of MRPS28 in cancer prognosis. In addition, MRPS28 expression had good diagnostic value in various cancers, especially in COAD, LIHC, LUSC, THYM, READ, TGCT, GBM, PAAD, LGG, and PCPG (AUC > 0.90). In BRCA, we found that the patients with higher expression of MRPS28 had poorer OS and DSS, and AUC > 0.8, which is consistent with previous reports (24). Taken together, these observations suggest that MRPS28 may serve as a prognostic and diagnostic biomarker for patients with cancer.

Genetic alterations were vital factors that contributed to tumor development and progression (25). In our study, genetic alterations in MRPS28 were analyzed in pan-cancer. The result indicated that the gene alteration types of MRPS28 were primary amplification and mutation. In BRCA, the genetic alterations of MRPS28 rank second among various cancers and are mainly amplified. Furthermore, tumor genomic heterogeneity plays a crucial role in determining the progression, metastasis, and treatment response of cancers (11, 12). Our analysis revealed significant correlations between the expression of MRPS28 and various genomic features, including TMB, MATH, MSI, NEO, PURITY, PLOIDY, HRD, and LOH. TMB and MSI may be used to predict the efficacy of immune checkpoint inhibitor drugs (26), and MSI was also a prognostic indicator and a predictor of treatment efficacy in some studies (27). We found that the expression of MRPS28 in BRCA is positively correlated with MATH, indicating that patients with high expression of MRPS28 have higher tumor heterogeneity, possibly worse prognosis, and increased treatment difficulty. MRPS28 is also negatively correlated with MSI in BRCA, indicating that high expression of MRPS28 is not conducive to patient prognosis or immunotherapy, etc. In addition, the expression of MRPS28 is significantly correlated with tumor purity, ploidy, and LOH, further illustrating the complex relationship between MRPS28 and genomic heterogeneity, providing a new target for the treatment of BRCA.

CSCs are known to promote tumor initiation, metastasis, and therapy resistance (28–30). Our results suggest that MRPS28 expression was positively correlated with several stemness indexes across various cancers including BRCA. This observation is supported by the fact that MRPS28 expression was also positively correlated with the DNA damage repair pathways, including MMR and HRR including BRCA, both of which are crucial for maintaining CSCs’ genomic integrity. These findings suggested that MRPS28 may affect the stemness and genomic stability of cancer cells, thereby promoting tumor progression. Methylation is a form of epigenetic regulation, and the methylation of DNA and RNA can regulate the expression of genes involved in cancer. Abnormal DNA methylation levels induce the occurrence and development of tumors by altering gene expression (31). Hypermethylation in the promoter region inhibits the expression of tumor suppressor genes (18). However, the hypomethylation contributes to the development of cancer by upregulating target gene expression (18). Our results reveal that MRPS28 methylation was significantly decreased in BLCA, BRCA, HNSC, LIHC, LUAD, LUSC, PRAD, READ, TGCT, and UCEC, whereas it was increased in KIRC and THCA. Correspondingly, patients with higher levels of MRPS28 methylation exhibited a good prognosis in BLCA, BRCA, HNSC, LIHC, LUAD, LUSC, READ, and UCEC, but this was reversed in KIRC. These results further suggest that MRPS28 hypomethylation promoted its expression in cancers, thereby leading to a poor prognosis.

Despite the great advances in treatment strategies, including surgical resection, chemotherapy, and radiotherapy, the prognosis of patients with cancer remains disappointing (32, 33). However, immunotherapies proposed in recent years exhibit promising results as cancer treatment strategies with great clinical application prospects, which are closely related to controlling the infiltration of immune cells or targeting immune checkpoints in the TME (34, 35). Immune cells are a key component of the tumor conditions. The infiltration of immune cells is substantially related to the tumor states (36, 37). Our results found that the expression of MRPS28 was closely correlated with the infiltration of activated CD8, memory CD8, Th1, Th2, Th17, Treg, activated B cells, memory B cells, immature B cells, monocytes, and NK T cells. Based on the different roles of these immune cells in tumors, the roles of MRPS28 in the tumor immune response may require consideration of the differences in the immune cells and tumor types, even though MRPS28 may be suitable for combination immunotherapy. Moreover, we found that multiple genes involved in immune checkpoint, chemokine, receptor, and MHC were associated with the expression of MRPS28. Thus, MRPS28 may be a potential target of cancer immunotherapy. In addition, Liu et al. found that MRPS28 expression was significantly correlated with immune cell infiltration, including naïve B cells, resting mast cells, CD4 memory T cells, T cells gamma delta, M1 macrophages, eosinophils, neutrophils, M2 macrophages, and memory B cells (5). Our study also evaluated the relationship between MRPS28 expression and infiltration scores by CIBERSORT. We found the more associations between MRPS28 and infiltration of immune cells in BRCA, but not in other cancers. Thus, our further analyses found that the expression of MRPS28 was positively correlated with the infiltration of M2 macrophage, resting NK cells, and activated mast cells. M2 macrophage promotes tumors by exerting immunosuppressive functions in various types of tumors (38). The increased infiltration of resting NK cells and activated mast cells indicates the formation of an immunosuppressive tumor microenvironment (39). But MRPS28 was negatively correlated with CD8^+^T cell, gamma delta T cell, follicular helper T cell, resting memory CD4^+^T cell, activated NK cell, resting myeloid dendritic cell, M1 macrophage, and naive B cells in BRCA, it is well known that these cells have anti-tumor effects. These results together indicated that increased MRPS28 expression may facilitate the formation of an immunosuppressive tumor microenvironment and lead to immune escape of tumor cells in BRCA.

Our multi-omics analyses revealed that MRPS28 expression is closely linked to immune cell infiltration, DNA damage repair pathways (MMR/HRR), cancer stemness indices, and epigenetic regulation via DNA methylation. These associations suggest that MRPS28 may exert its oncogenic effects through modulating the tumor immune microenvironment and maintaining genomic stability. Although the exact molecular mechanisms require further investigation, our data provide a strong foundation for these hypotheses. In terms of the findings in this study, we believe that the specific mechanism of MRPS28 regulating cancer progression may be related to the following five aspects (1). MRPS28 acts on mitochondrial metabolic reprogramming. Because MRPS28, as a mitochondrial ribosomal component, most likely affects the assembly of oxidative phosphorylation or other mitochondrial related component complexes by regulating the translation efficiency of mitochondrial encoded proteins, thus leading to the alteration of ATP/ROS balance and activating other downstream oncogenic signals. The GO-BP (**Figure 11D**) and KEGG (**Figure 11E**) enrichment analysis proved that the protein most related to MRPS28 was associated with translational elongation, mitochondrial gene expression, mitochondrial respiratory chain complex assembly, NADH dehydrogenase complex assembly, and oxidative phosphorylation have a strong correlation (2). MRPS28 regulates the formation of tumor immune microenvironment. MRPS28 may regulate the infiltration of M2 macrophages, CD8^+^T cells, or other immune cells by regulating the expression of chemokines or immune checkpoint molecules (**Figure 10**, **S1**) (3). MRPS28 regulates DNA damage repair and genome stability. Since MRPS28 is positively correlated with MMR/HRR gene expression (**Figures 7a, b**). Therefore, MRPS28 may affect the efficiency of homologous recombination or mismatch repair by interacting with DNA repair proteins such as MLH1 and MSH2, thus leading to enhanced genomic instability (4). MRPS28 exerts the mechanism of epigenetic regulation. MRPS28 expression is positively correlated with DNMTs, and its methylation level is correlated with patient prognosis (**Figure 8**). MRPS28 may lead to the change of promoter methylation status of specific genes by affecting the activity or localization of DNA methyltransferases (DNMTs) (5). MRPS28 contributes to the maintenance of cancer stem cell properties. We found that MRPS28 was positively correlated with multiple stemness indexes (**Figure 7C**).

Breast cancer is one of the most common cancers in women (40), and BRCA is a main subtype of breast cancer, which accounts for most cases of patients with breast cancer. BCRA has metastatic potential and a poor prognosis (41). At the same time, based on the above results that MRPS28 plays an important role in BRCA. Therefore, we focused on the role of MRPS28 in BRCA and confirmed the increased expression of MRPS28 in BRCA. MRPS28 expression was related to the subtype and lymph node metastasis status of BRCA. Importantly, MRPS28 expression was higher in patients with BRCA metastasis than in patients with non-metastatic BRCA. Correspondingly, we found that MRPS28 expression was positively correlated with cell invasion and metastasis by analyzing the relationship between MRPS28 expression and the functional state of breast cancer cells. It is known that metastasis is the most lethal cause of many types of cancer, including breast cancer (42, 43). Therefore, our findings indicate that MRPS28 plays an important role in BRCA and may be related to BRCA metastasis. In addition, we analyzed single-cell sequencing data related to BRCA, and we found that MRPS28 was highly expressed in different clusters. Furthermore, the MRPS28 expression levels were increased in CD4^+^T cells, CD8^+^T cells, and Treg cell clusters, which indicated that MRPS28 may regulate the infiltration of these immune cells in the BRCA TME, it was confirmed by the CIBERSORT scores results above. Breast cancer originates from breast epithelial cells (44, 45). Interestingly, we found abnormally high expression levels of MRPS28 in epithelial cell clusters, which indicates that MRPS28 may be related to the occurrence of breast cancer. Finally, we predicted 9 potential small molecule targeted drugs for MRPS28 through the “oncoPredict” R package, indicating that drug targeting MRPS28 has certain potential for cancer treatment.

Based on these results, we analyzed the effect of MRPS28 on the malignant biological behavior of breast cancer cells. We detected the MRPS28 protein expression in BRCA clinical specimens by IHC assay. The results revealed that MRPS28 expression was significantly increased in breast cancer tissues compared with the paired normal adjacent tissues. In addition, we found that MRPS28 knockdown significantly inhibited the proliferation, migration, and invasion and promoted the apoptosis of MCF-7 and MDA-MB-231 cells. These findings suggest that MRPS28 is a potential BRCA oncogene. While our results provides robust evidence for the oncogenic role of MRPS28 in breast cancer through comprehensive bioinformatics analyses and loss of function experiments *in vitro*, we acknowledge that further validation remains an important future direction *in vivo*. The consistency between results of bioinformatics analyses and MRPS28 expression in clinical samples (IHC) and its functional impact on proliferation, migration, invasion, and apoptosis in breast cancer cell lines strongly supports its potential as a therapeutic target. In the future, studies utilizing patient-derived xenograft (PDX) models or transgenic mouse models will be essential to confirm the tumor-promoting effects and mechanisms of MRPS28 in BRCA *in vivo*.

## Conclusions

In conclusion, this study performed a comprehensive analysis of the roles of MRPS28 in pan-cancer, especially in BRCA. Our study indicated that MRPS28 may be a potential diagnostic, prognostic, and immunotherapy-related marker for various malignancies. The high expression of MRPS28 was significantly associated with a poor prognosis in patients and promoted the malignant biological behavior of breast cancer cells. However, this study has some limitations. First, we did not explore the effect of MRPS28 on the tumor growth of BRCA *in vivo*. The regulatory mechanisms of MRPS28 in BRCA progression need to be investigated further. Future work should also focus on elucidating the exact molecular mechanisms through which MRPS28 exerts its oncogenic effects, particularly its role in mitochondrial-immune crosstalk, DNA damage response, and epigenetic regulation *in vivo* and *in vitro*. In addition, in the future, we should construct multi omics knowledge graphs and use deep learning such as graph convolutional networks (GCNs) for cross modal association prediction. Combined with pathological images and gene expression data, multimodal GCN was used to model the prognosis of breast cancer (46).

## Data Availability

The original contributions presented in the study are included in the article/**Supplementary Material**. Further inquiries can be directed to the corresponding authors.

## Supplementary material

Supplementary Figure 1 Analysis of the relationship between MRPS28 expression and infiltration scores. The relationship between MRPS28 expression and infiltration scores in pan-cancer was evaluated using CIBERSORT R package. *p < 0.05, **p < 0.01, and ***p < 0.001.

Supplementary Figure 2 Drug sensitivity analysis of MRPS28. Drug sensitivity analysis was performed using the “oncoPredict” R package. According to the ranking of correlation coefficient values from high to low, 9 drugs were displayed.
