# Supplementary figures and images for: MRPS28 serves as a biomarker of diagnostic, prognostic, and immune modulation in pan-cancer and promotes breast cancer malignant phenotypes
Source: Front Immunol. 2026 Mar 3;17:1680772. doi: 10.3389/fimmu.2026.1680772 (PMC12992041; doi:10.3389/fimmu.2026.1680772)

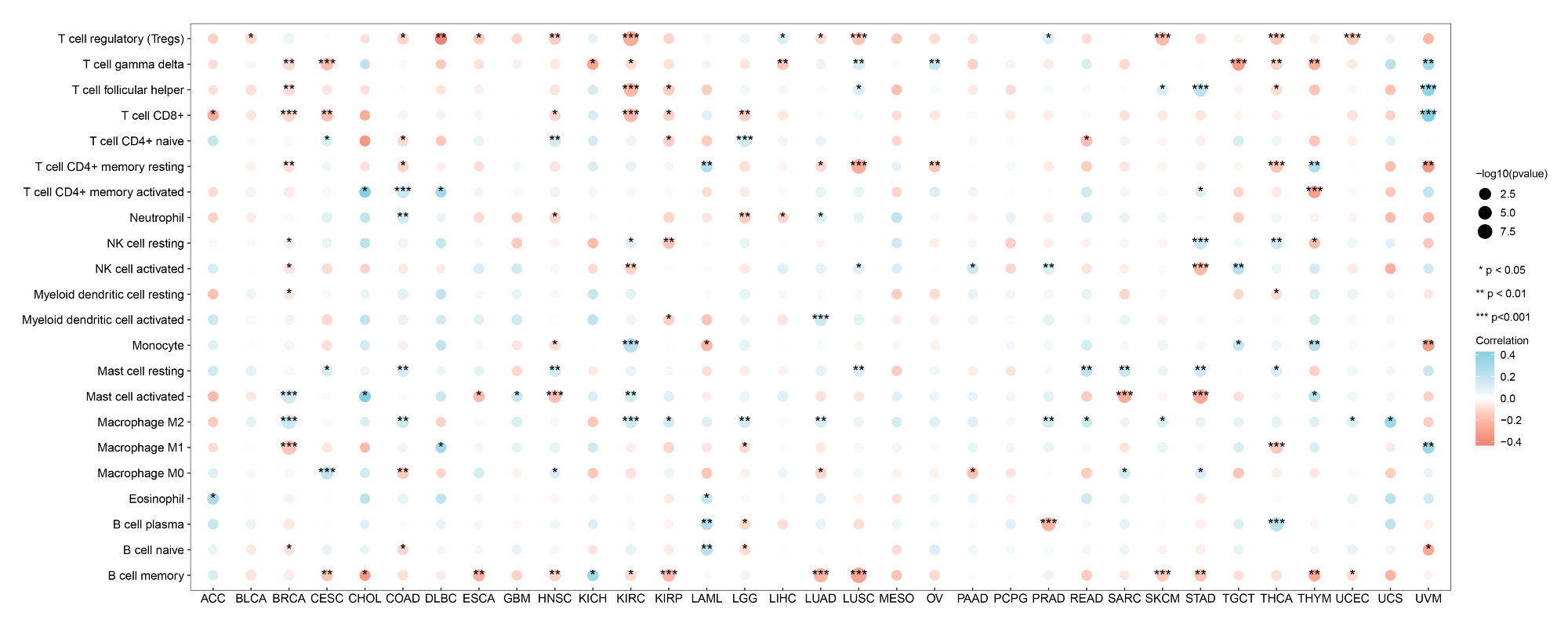

Supplement: Supplementary Figure 1 — Analysis of the relationship between MRPS28 expression and infiltration scores. The relationship between MRPS28 expression and infiltration scores in pan-cancer was evaluated using CIBERSORT R package. *p < 0.05, **p < 0.01, and ***p < 0.001. [file Image1.tif]

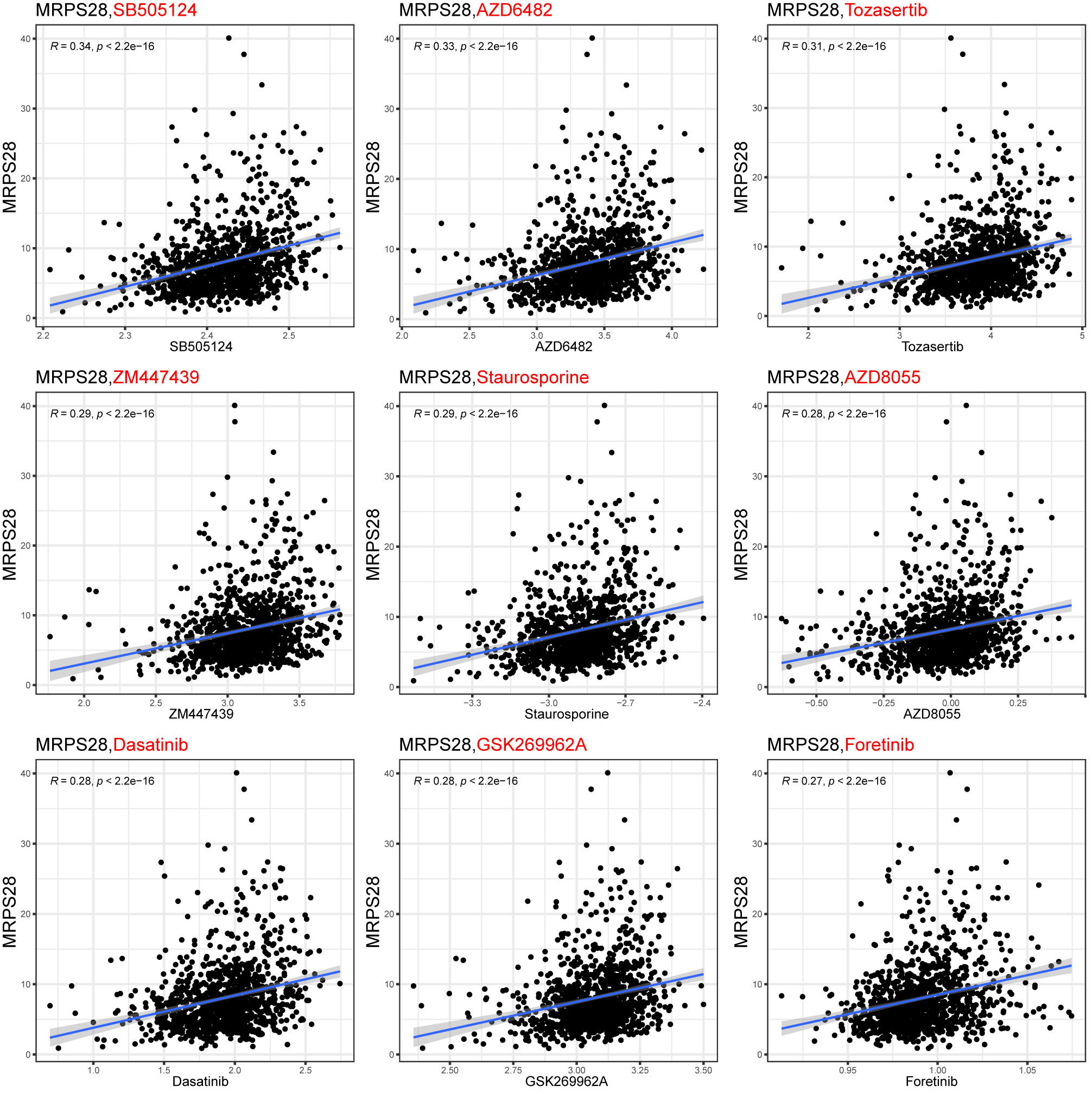

Supplement: Supplementary Figure 2 — Drug sensitivity analysis of MRPS28. Drug sensitivity analysis was performed using the “oncoPredict” R package. According to the ranking of correlation coefficient values from high to low, 9 drugs were displayed. [file Image2.tif]

Figure 13B

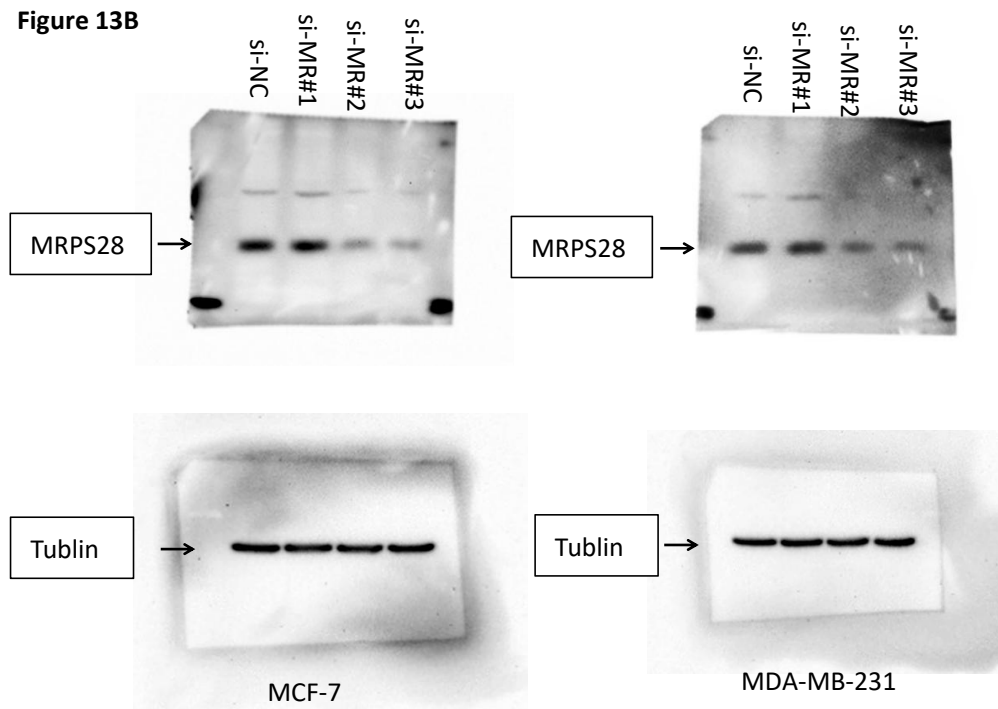

Supplement: Supplementary file 3 [file DataSheet1.pdf]
