# Supplementary material for: MRPS28 serves as a biomarker of diagnostic, prognostic, and immune modulation in pan-cancer and promotes breast cancer malignant phenotypes
Source: Front Immunol. 2026 Mar 3;17:1680772. doi: 10.3389/fimmu.2026.1680772 (PMC12992041; doi:10.3389/fimmu.2026.1680772)
Supplement: Supplementary file 4 [file Table1.docx]

| **Abbreviation** | **Full name** | **Data source** |
| --- | --- | --- |
| **ACC** | Adrenocortical carcinoma | TCGA |
| **BLCA** | Bladder urothelial carcinoma |  |
| **BRCA** | Breast invasive carcinoma |  |
| **CESC** | Cervical squamous cell carcinoma and endocervical adenocarcinoma |  |
| **CHOL** | Cholangiocarcinoma |  |
| **COAD** | Colon adenocarcinoma |  |
| **DLBC** | Lymphoid neoplasm diffuse large B-cell lymphoma |  |
| **ESCA** | Esophageal carcinoma |  |
| **GBM** | Glioblastoma multiforme |  |
| **GBMLGG** | Glioblastoma and Lower Grade Glioma |  |
| **HNSC** | Head and neck squamous cell carcinoma |  |
| **KICH** | Kidney chromophobe |  |
| **KIRC** | Kidney renal clear cell carcinoma |  |
| **KIRP** | Kidney renal papillary cell carcinoma |  |
| **KIPAN** | **Pan-Kidney cohort** |  |
| **LAML** | Acute myeloid leukemia |  |
| **LGG** | Lower grade glioma |  |
| **LIHC** | Liver hepatocellular carcinoma |  |
| **LUAD** | Lung adenocarcinoma |  |
| **LUSC** | Lung squamous cell carcinoma |  |
| **MESO** | Mesothelioma |  |
| **OV** | Ovarian serous cystadenocarcinoma |  |
| **PAAD** | Pancreatic adenocarcinoma |  |
| **PCPG** | Pheochromocytoma and paraganglioma | TCGA |
| **PRAD** | Prostate adenocarcinoma |  |
| **READ** | Rectum adenocarcinoma |  |
| **SARC** | Sarcoma |  |
| **SKCM** | Skin cutaneous melanoma |  |
| **STAD** | Stomach adenocarcinoma |  |
| STES | Stomach and esophageal sarcinoma |  |
| TGCT | Testicular germ cell tumor |  |
| **THCA** | Thyroid carcinoma |  |
| **THYM** | Thymoma |  |
| **UCEC** | Uterine corpus endometrial carcinoma |  |
| **UCS** | Uterine carcinosarcoma |  |
| **UVM** | Uveal melanoma |  |
| ccRCC | Clear Cell Renal Cell Carcinoma |  |
| WT | Wilms Tumor | Therapeutically Applicable Research to Generate Effective Treatments (TARGET) |
| ALL | Acute Lymphoblastic Leukemia |  |
| RECA | Renal cancer | The Human Protein Atlas |
